# Early Warning Signals of Financial Crises with Multi-Scale Quantile Regressions of Log-Periodic Power Law Singularities

**DOI:** 10.1371/journal.pone.0165819

**Published:** 2016-11-02

**Authors:** Qun Zhang, Qunzhi Zhang, Didier Sornette

**Affiliations:** 1 School of Finance, Guangdong University of Foreign Studies, Guangzhou, Guangdong, China; 2 Department of Management, Technology and Economics, ETH Zurich (Eidgenössische Technische Hochschule Zürich), Zurich, Switzerland; 3 Swiss Finance Institute, c/o University of Geneva, Geneva, Switzerland; Tianjin University, CHINA

## Abstract

We augment the existing literature using the Log-Periodic Power Law Singular (LPPLS) structures in the log-price dynamics to diagnose financial bubbles by providing three main innovations. First, we introduce the quantile regression to the LPPLS detection problem. This allows us to disentangle (at least partially) the genuine LPPLS signal and the a priori unknown complicated residuals. Second, we propose to combine the many quantile regressions with a multi-scale analysis, which aggregates and consolidates the obtained ensembles of scenarios. Third, we define and implement the so-called DS LPPLS Confidence^™^ and Trust^™^ indicators that enrich considerably the diagnostic of bubbles. Using a detailed study of the “S&P 500 1987” bubble and presenting analyses of 16 historical bubbles, we show that the quantile regression of LPPLS signals contributes useful early warning signals. The comparison between the constructed signals and the price development in these 16 historical bubbles demonstrates their significant predictive ability around the real critical time when the burst/rally occurs.

## Introduction

The daily actions resulting from the entangled interactions between investors in markets with ever more numerous financial innovations are the cause of the increasingly inherent complexity of price dynamics. This complexity is revealed through the occurrence of varied market regimes, from transient bubbles, to high volatility markets and prolonged market negative performance. The present theoretical knowledge and empirical methodologies are insufficient to fully capture the emerging risks. As financial markets provide both a measure of the health of the underlying economy and an engine for funding firms and catalysing growth, it is urgent to develop new approaches to describe the large price fluctuations and to develop testable diagnostics of financial bubbles. The present article aims at extending the approach pioneered in [1–6] to develop novel testable diagnostics of financial bubbles. Real time monitoring and timely early warning of finance bubbles are not only an important part of recent academic research to expand on the efficient market hypothesis. They are also motivated by concrete real life applications to possibly avoid financial crises and at least prepare against them to ensure a prompt and efficient response [7–9]. Various scientific platforms have been built to monitor asset prices and to study financial bubbles. Here, we build on the Financial Crisis Observatory at ETH Zurich (http://www.er.ethz.ch/financial-crisis-observatory.html), which has the goal of testing rigorously the hypothesis that financial markets exhibit a degree of inefficiency and a potential for predictability, especially during regimes when bubbles develop.

In general, normal times are characterised by an approximate constant return (or price growth rate). This is nothing but the statement that the average price trajectory is a noisy exponential that reflects the power of compounding interests. As the simplest embodiment of this noisy exponential growth, the Geometrical Brownian Motion model is the starting point of more sophisticated models in financial mathematics and financial engineering. However, financial markets often deviate strongly from such simple description in the form of bubbles, defined as periods in which asset prices strongly deviate from the corresponding fundamental value. One of the practical problems of bubble identification is that the fundamental value is not directly observable and is roughly estimated within a factor of 2 [10], typically. Based on the analyses of many historical bubbles, the studies [1–3, 11] have documented that there are transient regimes during which the price growth rate (return) grows itself, which translates into a super-exponential time dynamics. Such a procyclical process involving positive feedbacks, which can be of many types, such as option hedging, portfolio insurance strategies, margin requirements, as well as the imitation and herding behavior in psychology. These mechanisms tend to increase and accelerate the deviation from an equilibrium. The resulting super-exponential price trajectories are inherently unsustainable and often burst as crashes or strong corrections. In a nutshell, the existence of a transient faster-than-exponential price growth can be taken as a signature of bubbles [6, 11, 12]. The advantage of this definition of a bubble is that it does not rely on the estimation of what is a fundamental value (see e.g., [13]), which is poorly known as mentioned above.

The Log-Periodic Power Law Singularity (LPPLS) model has been proposed as a simple generic parameterisation to capture such super-exponential behavior [1–4], which is inspired from physics (and is sometimes referred to as part of econophysics [14]). This model takes into account that positive feedbacks generically lead to finite-time singularities [9, 15, 16]. Moreover, it includes log-periodic oscillations decorated by accelerating oscillations, which are the observable embodiment of the symmetry of discrete scale invariance [17]. This generic log-periodicity accounts for the existence of a discrete hierarchy of group sizes [18] and may also result from the interplay between nonlinear value investors and nonlinear trend followers, and the inertia between information flow and price discovery [15]. In summary, the LPPLS model provides a convenient representation of financial bubbles.

As mentioned above, the LPPLS model is the simplest analytical formulation of time series that possess a discrete regular hierarchy of time scales [17]. It is a particularly useful tool among the large set of concepts and methods dealing with multi-scale analysis of mono- and multi-variate time series, which include temporal multifractal analysis [19–22], directed weighted network representations of time series using the delayed coordinate embedding method combined with a distance that provides an adjacency matrix [23–26], and a variety of techniques at the intersection of nonlinear dynamical system theory, statistical time series analysis, fractals, cellular automata, machine learning methods, wavelet transform methods, fuzzy logic and more [27, 28].

We thus follow up on these previous efforts to diagnose financial bubbles and their terminations by proposing several innovations. First, rather than using the standard least squares or maximum likelihood calibration method, we apply the quantile regression method to the LPPLS calibration problem. In other words, rather than fitting a given log-price time series by a single LPPLS model, quantile regressions provide a family of calibrated curves indexed by the probability level *q*. Scanning *q* between 0 and 1 allows us to disentangle (at least partially) the genuine LPPLS signal from the a priori unknown complicated residuals. Moreover, this new technology alleviates some of the statistical problems that have plagued the literature: error in variables, sensitivity to outlier and non-normal error distributions [29]. It provides a descriptive approach reporting more than just the expected mean of a conditional distribution, but may also discover more complete structures without imposing global distributional assumptions on the residuals. In contrast, the standard least squares or maximum likelihood estimation procedures are vulnerable to the existence of outliers [30]. In sum, the prediction inference associated with quantile-based estimates has an inherent distribution-free character since they are influenced only by the local behavior of the underlying distribution near the specified quantile [31]. The different *q*-dependent LPPLS fits also provide a bundle of possible scenarios that are compatible with different weights of the residuals supposed to decorate the theoretical driver.

While the implementation of ensemble forecasting from quantile estimates is still in its infancy, we apply the ensemble forecasting obtained from the quantile regressions at various *q* values to construct early warning signals. This is proposed to improve on the common practice of relying on one single calibration to make forecasts. This provides a representative sample of the possible future states in order to improve generalization and robustness compared with single estimators [32]. On average, the combined estimator is usually better than any of the single base estimator because its variance is reduced. The median of individual estimates is more accurate than at least half of the individual forecasts [33].

Then, we propose to combine the many quantile regressions with a multi-scale analysis. This leads to the development of ensemble forecasting that combines a grid of quantile-based estimators into a final aggregated predictor. We further introduce the Quantile-Violin plots and the *dt*-Violin plots as powerful representations of the enormous amount of information generated by scanning the quantile levels and the time scales.

Finally, we define and implement the so-called DS LPPLS Confidence^™^ and Trust^™^ indicators, which provide an aggregation and consolidation of the wealth of generated information and we put them at work to diagnose 16 historical bubble cases. Positive bubbles and negative bubbles can be respectively identified from the performance of these systemic indicators.

We proceed as follows. Section 2 presents the LPPLS model and gives an overview on some theoretical aspects of the standard ordinary least square regression (referred to as the *L*^2^ norm calibration) and of quantile regressions. Section 3 presents the metrics, the methodology and a battery of tests performed on the S&P 500 bubble that burst in October 1987. In particular, we introduce the Quantile-Violin plots and the *dt*-Violon plots as efficient presentations of the multi dimensional metrics. Section 4 extends section 3 to three other historical financial bubbles, providing interesting elements of comparison. Section 5 introduces the DS LPPLS Confidence^™^ and Trust^™^ indicators. Section 6 applies all the above tools and metrics to 16 historical financial bubbles and compare the indicators with the price time series. Section 7 summarises our main conclusions.

## Model and calibrations

### Log-Periodic Power Law Singularity (LPPLS) model

The Johansen-Ledoit-Sornette (JLS) model [2, 3] assumes that the asset price *p*(*t*) follows a standard diffusive dynamics with varying drift *μ*(*t*) in the presence of discrete discontinuous jumps:

$$\begin{array}{c} \frac{dp}{p}=\mu \left(t\right)dt+\sigma \left(t\right)dW-\kappa dj, \end{array}$$ (1)

where *σ*(*t*) is the volatility and *dW* is the increment of a Wiener process (with zero mean and variance equal to *dt*). The term *dj* represents a discontinuous jump such that *j* = 0 before the crash and *j* = 1 after the crash occurs. The loss amplitude associated with the occurrence of a crash is determined by the parameter *κ*. Each successive crash corresponds to a jump of *j* by one unit. The dynamics of the jumps is governed by a crash hazard rate *h*(*t*). Since *h*(*t*)*dt* is the probability that the crash occurs between *t* and *t* + *dt* conditional on the fact that it has not yet happened, we therefore have the expectation *E*_*t*_[*dj*] = 1 × *h*(*t*)*dt* + 0 × (1 − *h*(*t*))*dt* = *h*(*t*)*dt*. By the no-arbitrage condition leading to the condition that the price process is a martingale ($E_{t}[\frac{dp}{p}]=0$, neglecting the risk free rate), it leads to *μ*(*t*) = *κh*(*t*).

Under the assumption of the JLS model, the crash hazard rate aggregated by the noise traders with herding behaviors has the following dynamics:

$$\begin{array}{c} h\left(t\right)\approx B_{0}{|t_{c}-t|}^{m-1}+C_{0}{|t_{c}-t|}^{m-1}\cos(\omega \ln|t_{c}-t\left|+\phi^{\prime }\right). \end{array}$$ (2)

Using *μ*(*t*) = *κh*(*t*), we obtain the dynamics of the expectation of the logarithm of the price in the form of the Log-Periodic Power Law Singularity (LPPLS) model:

$$\begin{array}{c} E\left[\ln p\left(t\right)\right]=A+B{|t_{c}-t|}^{m}+C{|t_{c}-t|}^{m}\cos(\omega \ln|t_{c}-t\left|+\phi \right), \end{array}$$ (3)

where *t*_*c*_ denotes the most probable time for the burst of the bubble, in the form of a crash for example. The constant *A* = ln[*p*(*t*_*c*_)] gives the terminal log-price at the critical time *t*_*c*_. $B=sgn\left(t-t_{c}\right)\frac{\kappa B_{0}}{m}$ and $C=sgn\left(t-t_{c}\right)\frac{\kappa C_{0}}{\sqrt{m^{2}+\omega^{2}}}$ respectively control the amplitude of the power law acceleration and of the log-periodic oscillations. The exponent *m* quantifies the degree of super-exponential growth. The log-periodic angular frequency *ω* is related to a scaling ratio $\lambda =\exp(\frac{2\pi }{\omega })$ of the temporal hierarchy of accelerating oscillations converging to *t*_*c*_. Finally, *ϕ* ∈ (0, 2*π*) is a phase embodying a characteristic time scale of the oscillations. Eq (3) is the first-order log-periodic correction to a pure power law for an observable exhibiting a singularity at *t*_*c*_ [4, 34].

Given the starting and ending dates *t*_*start*_ and *t*_*end*_ of the fitting window, we define $dt≜t_{end}-t_{start}$ as the duration of the fitting window. The critical time *t*_*c*_ is searched in the interval [*t*_*end*_ − *ηdt*, *t*_*end*_ + *ηdt*], with *η* is typically equal to 0.20. Previous calibrations of the LPPLS specification Eq (3) to the log-price development during a number of historical financial bubbles have suggested to qualify fits based on the parameters of the LPPLS model belonging to the following intervals [5, 35, 36]: *m* ∈ [0.1, 0.9], *ω* ∈ [6, 13], |*C*| ≤ 1, *B* < 0. In our explorations, we have found that relaxing the search space to the larger intervals *m* ∈ [0, 2] and *ω* ∈ [1, 50] does not change the results significantly, particularly for the calibrated critical times within statistical fluctuations. The results we report below have thus been obtained for the larger search ranges *m* ∈ [0, 2], *ω* ∈ [1, 50].

### The optimization problem using the standard Ordinary Least Squares (OLS) method

Filimonov and Sornette [36] suggested to expand the cosine term of Eq (3) with *C*_1_ = *C* cos *ϕ*, *C*_2_ = −*C* sin *ϕ* to obtain a representation with 4 linear and 3 nonlinear parameters, providing a substantial gain in efficiency and stability of the calibration. This leads to rewrite Eq (3) as

$$\begin{array}{c} \ln E\left[p\left(t\right)\right]=A+B{|t_{c}-t|}^{m}+C_{1}{|t_{c}-t|}^{m}\cos(\omega \ln|t_{c}-t|)+C_{2}{|t_{c}-t|}^{m}\sin(\omega \ln|t_{c}-t\left|\right). \end{array}$$ (4)

The optimization problem with the standard Ordinary Least Squares (OLS) method aims to minimize the sum *F*(*t*_*c*_, *m*, *ω*, *A*, *B*, *C*_1_, *C*_2_) of squared residuals between the log-price ln *p*(*t*_*i*_), *i* = 1, 2, …, *N* and Eq (4), where

$$\begin{array}{ccc} & & F(t_{c},m,\omega ,A,B,C_{1},C_{2})\\ & & \;=\sum_{i=1}^{N}(\ln p\left(t_{i}\right)-A-B{|t_{c}-t_{i}|}^{m}-C_{1}{|t_{c}-t_{i}|}^{m}\cos(\omega \ln|t_{c}-t_{i}\left|\right)\\ & & \;-\;{C_{2}{|t_{c}-t_{i}|}^{m}\sin(\omega \ln|t_{c}-t_{i}\left|)\right)}^{2}\;. \end{array}$$ (5)

### The optimization problem using the Quantile Regression calibration method

Intuitively, the OLS calibration method is finding the best fit “in mean”. In other words, the parameters are adjusted so that the function to calibrate is the closest to the mean of the noisy realisation of the log-price, where the mean should be considered conceptually to occur over many realisations of the noise decorating the supposed theoretical function Eq (4). If the noise is not normally distributed and exhibits heavier tails, the OLS calibration may be contaminated by large deviations of the noise from the mean. Then, fitting the data to the function that is the closest to the median of the noisy realisation of the log-price may be more adequate and lead to more stable estimations. It is well known that this amounts to replacing the *L*^2^ norm (sum of the square of the differences) in Eq (5) by the *L*^1^ norm (sum of the absolute value of the differences). Quantile regressions amount to generalizing the minimisation of the *L*^1^ norm and provide not just a single best fit to the median but a bundle of best fits to the different quantile realisations of the noise around the theoretical LPPLS function Eq (4).

First, let us recall that the *q*th quantile of a random variable *Y* with distribution function *F*_*Y*_(*y*) = *P*(*Y* ≤ *y*) is defined as

$$\begin{array}{c} Q_{Y}\left(q\right)=\inf\left\{y|F_{Y}\left(y\right)\geq q,\;q\in \left(0,1\right)\right\}\;. \end{array}$$ (6)

Let us define the *q*-dependent loss function with respect to residual *e*_*t*_:

$$\begin{array}{c} \rho_{q}\left(e_{t}\right)=\{\begin{array}{cc} -\left(1-q\right)e_{t} & if\;e_{t}<0,\\ qe_{t} & if\;e_{t}\geq 0. \end{array} \end{array}$$ (7)

For *q* = 1/2, $\rho_{1/2}\left(e_{t}\right)=\frac{1}{2}\left|e_{t}\right|$, so minimizing *ρ*_1/2_(*e*_*t*_) is nothing but minimising the *L*^1^ norm.

Quantile regression corresponds to finding the quantile-dependent parameters $\{\hat{t_{c}}\left(q\right)$, $\hat{m}\left(q\right)$, $\hat{\omega }\left(q\right)$, $\hat{A}\left(q\right)$, $\hat{B}\left(q\right)$, $\hat{C_{1}}\left(q\right)$, $\hat{C_{2}}\left(q\right)\}$ that minimise the function

$$\begin{array}{ccc} q(t_{c},m,\omega ,A,B,C_{1}{,}_{2}) & = & \sum_{i=1}^{N}\rho_{q}\{\ln p\left(t_{i}\right)-A-B{|t_{c}-t_{i}|}^{m}-\\ & & C_{1}{|t_{c}-t_{i}|}^{m}\cos(\omega \ln|t_{c}-t_{i}\left|\right)-C_{2}{|t_{c}-t_{i}|}^{m}\sin(\omega \ln|t_{c}-t_{i}|)\}. \end{array}$$ (8)

In other words, for each quantile level *q*, we obtain a set of *q*-dependent calibrated parameters

$$\begin{array}{c} \left\{\hat{t_{c}}\left(q\right),\hat{m}\left(q\right),\hat{\omega }\left(q\right),\hat{A}\left(q\right),\hat{B}\left(q\right),\hat{C_{1}}\left(q\right),\hat{C_{2}}\left(q\right)\right\}=\underset{t_{c},m,\omega ,A,B,C_{1},C_{2}}{\text{argmin}}S_{q}\left(t_{c},m,\omega ,A,B,C_{1},C_{2}\right)\;. \end{array}$$ (9)

To significantly decrease the complexity of the search and provide an intuitive representation of the results of the calibration, a two-stage fitting procedure is developed according to the special structure of the LPPLS model [36]. That is, according to Eq (4), the complexity of the optimization problem is reduced by slaving 4 linear parameters to the 3 nonlinear parameters. In essence, for minimizing the objective function of the OLS or Quantile Regressions, the linear parameters {*A*, *B*, *C*_1_, *C*_2_} or {*A*(*q*), *B*(*q*), *C*_1_(*q*), *C*_2_(*q*)} are determined using the LU decomposition algorithm through a linear regression model, while the nonlinear parameters {*t*_*c*_, *m*, *ω*} or {*t*_*c*_(*q*), *m*(*q*), *ω*(*q*)} are searched globally through the Taboo search followed by the Quasi-Newton method with line search.

From the definition in Eqs (7) and (8), one can see that the quantile regression is an asymmetrically weighted *L*^1^-based regression, where the asymmetry is governed by the value *q*. The special case *q* = 1/2 is symmetric and recovers the aforementioned *L*^1^ norm calibration. For *q* ≠ 1/2, by construction of Eq (7), the best fit corresponds statistically to *q* ⋅ 100% of the data points {ln *p*(*t*_*i*_), *i* = 1, 2, …, *N*} to be below the theoretical curve $\hat{\ln p_{q}\left(t\right)}$ and (1 − *q*) ⋅ 100% of the data points to be above it. Thus, for *q* > 1/2 (resp. *q* < 1/2), most of the data points are below (resp. above) the calibrated curve $\hat{\ln p_{q}\left(t\right)}$, putting it above (resp. below) the median fit.

## Methodology, metrics and tests on “S&P 500 1987” bubble

To illustrate the performance of the OLS and quantile regression methods, we test them on the time series of the S&P 500 Composite Index over the time period corresponding to the bubble that burst with the crash in October 1987, hereafter referred to as the “S&P 500 1987” bubble.

### LPPLS quantile regression curves for different quantile probability level *q*

**Fig 1:**
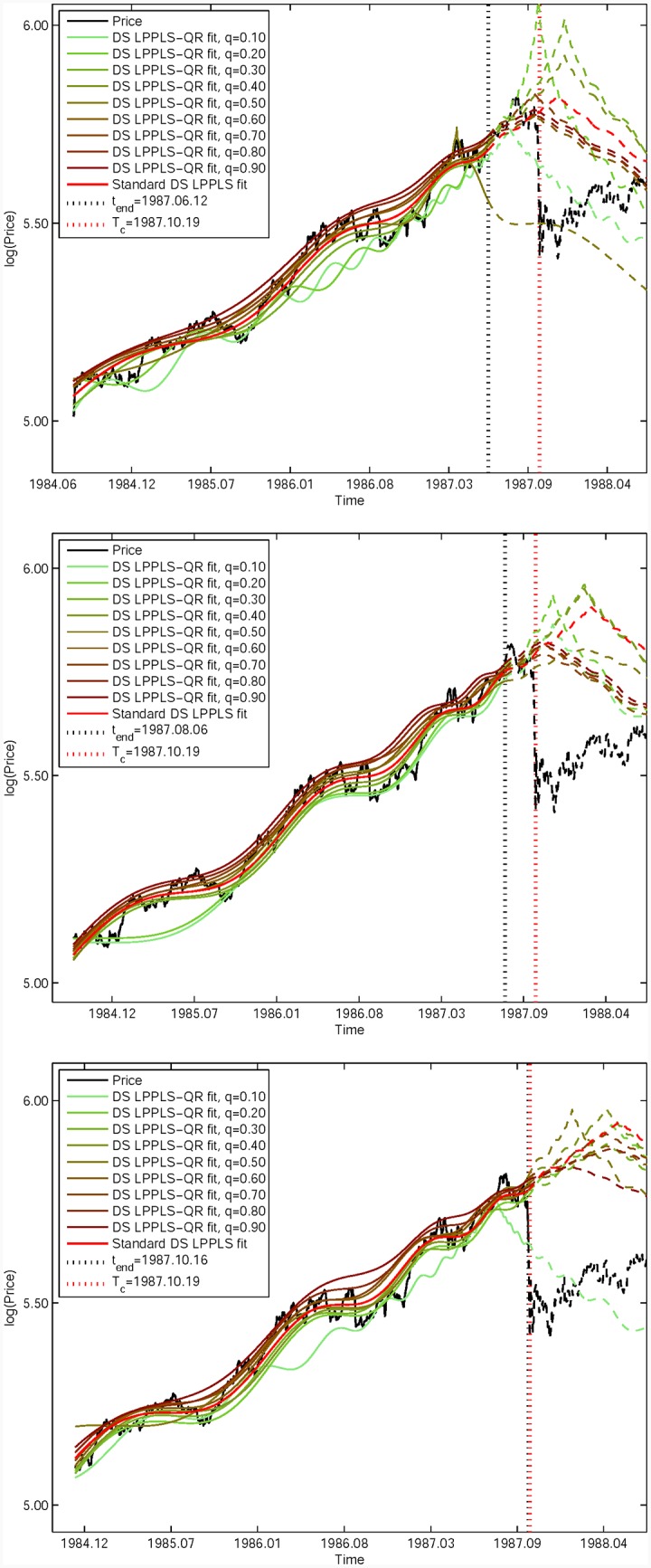
Nine coloured calibrated curves obtained by the method of LPPLS quantile regression for nine different *q* of the “S&P 500 1987” bubble. (A) Curves in the time window [1984.07.30, 1987.06.12]. (B) Curves in [1984.09.21, 1987.08.06]. (C) Curves in [1984.12.03, 1987.10.16]. The in-sample continuous curves in each panel are extended by dashed out-of-sample lines with the same colours. The noisy black line is the in-sample empirical price time series, followed by the black dashed out-of-sample data. The red thick line is the standard *L*^2^-based fitting curve for comparison, which is extended by the red dashed out-of-sample curve. The black dashed vertical line shows the value of *t*_*end*_ used in the calibration. The red dashed vertical line gives the true *T*_*c*_ = 1987.10.19.

Fig 1 represents a bundle of nine coloured quantile-based calibrated curves obeying expression Eq (4) obtained using the quantile regression method Eq (8) with Eq (7)) for nine quantile probability level *q* = 0.10, 0.20, …, 0.90. The three panels correspond to three time windows [1984.07.30, 1987.06.12] (top panel), [1984.09.21, 1987.08.06] (middle panel) and [1984.12.03, 1987.10.16] (bottom panel). The black dashed vertical line in each panel represents the corresponding end date *t*_*end*_ of the in-sample window. The red dashed vertical line is the true critical date *T*_*c*_ = 1987.10.19. The in-sample standard *L*^2^-based fitted curve is also shown as the red thick curve, which is extended by the red dashed thick out-of-sample curve.

From the three panels, one can see that the quantile curves cover approximately 80% of the variability of the empirical price time series, as they should according to the choice of *q* spanning from 0.10 to 0.90. The smaller (resp. larger) values of *q* tend to fit the lowest (resp. highest) part of the time series, providing together fuzzy envelops of the time series that seem quite reasonable visually. Note that these estimated critical times $\hat{t_{c}}$ correspond to the times at which the calibrated curves peak. For the Fig 1A and 1B corresponding to *t*_*end*_ not to close from the crash, one can observe that, apart from the lowest quantiles that exhibit more variability, the higher values of the quantiles provide consistent fits with estimated values of the critical time $\hat{t_{c}}$ close to the true value *T*_*c*_. In contrast, the standard *L*^2^-based fit tends to overshoot, similarly to the lowest quantiles. The situation reverses for the Fig 1C with *t*_*end*_ being very close to the crash, for which most of the quantiles (and the *L*^2^-based fit) overshoot significantly by about five months, while the lowest curve for *q* = 0.10 undershoots by approximately two months.

The divergence between the fitted functions obtained for low *q*’s and large *q*’s illustrates the first advantage of quantile regressions for LPPLS signals, that is, to provide a range of possible scenarios that can bracket the true value of *T*_*c*_, given that scanning *q* provides a family of calibrated functions that are sensitive to different parts of the statistical fluctuations supposed to decorate the theoretical generating process in Eq (4). More generally, one never knows precisely how the noise entangles with the LPPLS signals. Practical scenarios are more challenging in that the data often have unequal variation (a “location-scale model” in statistical terminology) due to the complex interactions between the various factors. This implicitly recognizes that there might be not a single super-exponential rate of change that characterizes changes in the probability distribution of log-price. In such cases, as well as in the presence of model errors (the true generating process is not known and the LPPLS model is only an approximation), quantile regressions provide a useful reading of the influence of the different noise quantile levels on the calibration results. The quantile regression also allows one to explore the heterogeneity of residuals as a function of time and deals with the asymmetric shape of the conditional distribution, which might be missed by OLS regression.

### Multi-scale analysis of $\hat{t_{c}}$ as a function of *q* and *dt*

#### $\hat{t_{c}}\left(q,dt\right)$ versus *t*_*end*_

In a real time situation, *t*_*end*_ of the time window corresponds to the last time at which data is available to perform the analysis. Considering a potential bubble bursting at the true critical time *T*_*c*_, *t*_*end*_ is the “present” time up to which the LPPLS signal is available, from which an estimation of the bubble end time $\hat{t_{c}}$ can be formed. There is an inherent tradeoff among these three times *t*_*end*_, *T*_*c*_ and $\hat{t_{c}}$. When *t*_*end*_ is far from *T*_*c*_, it is unlikely that the existing information is rich enough to provide an accurate prediction $\hat{t_{c}}$. Conversely, when *t*_*end*_ is close to *T*_*c*_, the singular nature of the LPPLS trajectory makes the determination $\hat{t_{c}}$ sensitive to the idiosyncratic realisation of the noise.

**Fig 2:**
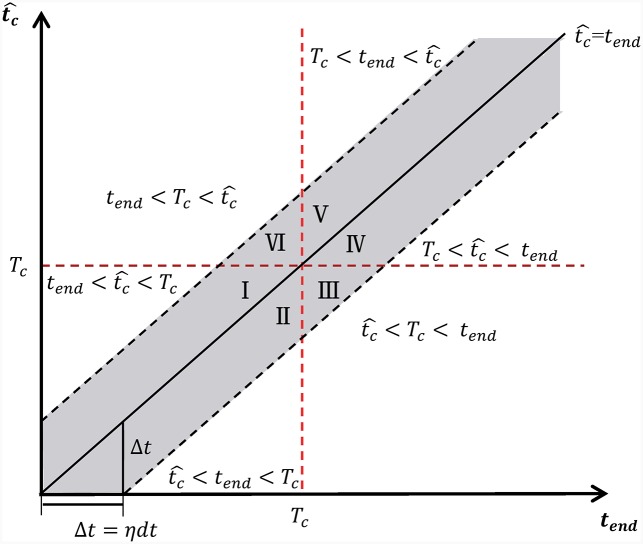
$t_{end}-\hat{t_{c}}$ plane. The six possible regimes associated with the different relationships between *t*_*end*_, *T*_*c*_ and $\hat{t_{c}}$ are depicted. The true time *T*_*c*_ at which the bubble bursts is indicated by the red dashed horizontal and vertical lines.

It is thus necessary to study their relationships systematically. We introduce the $t_{end}-\hat{t_{c}}$ plane as shown in Fig 2, in which *T*_*c*_ is indicated by the red dashed horizontal and vertical lines. The black diagonal line $\hat{t_{c}}=t_{end}$ separates the region where the estimated burst is in the future ($\hat{t_{c}}>t_{end}$, domain above the diagonal) from the region where the estimated burst is in the past ($\hat{t_{c}}<t_{end}$, domain below the diagonal). The grey band represents the searched interval [*t*_*end*_ − *ηdt*, *t*_*end*_ + *ηdt*] of $\hat{t_{c}}$ in the calibration, as explained in section 2. Then, one can identify six possible regions (represented by the roman numbers I to VI) associated with the different relationships among *t*_*end*_, *T*_*c*_ and $\hat{t_{c}}$.

- **Regions I and VI (True Positives)** are the most desired situations in which the bubble is on-going and the predicted $\hat{t_{c}}$ is in the future.
- **Region II (False Negatives)** corresponds to a failure of the prediction that purports that the bubble has ended ($\hat{t_{c}}<t_{end}$), while this is not true (*T*_*c*_ > *t*_*end*_).
- **Regions III and IV (True Negatives)** represent the case where the bubble has already ended and the calibration correctly diagnoses it.
- **Region V (False Positives)** is another failure of the prediction, which is opposite to region II. The prediction is that the bubble continues and its critical time $\hat{t_{c}}$ is in the future ($\hat{t_{c}}>t_{end}$), while it has truly ended (*T*_*c*_ < *t*_*end*_).

**Fig 3:**
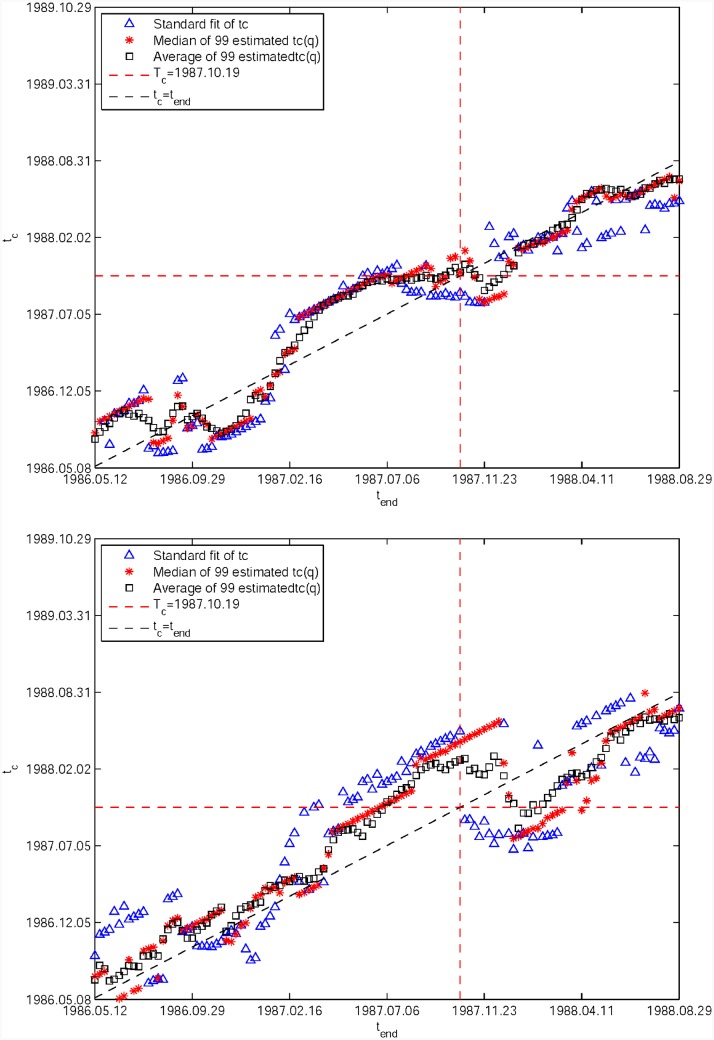
Average and median predictions of $\hat{t_{c}}\left(q,dt\right)$ as functions of *t*_*end*_ for two different window size. (A) *dt* = 500 trading days. (B) *dt* = 750 trading days. $\hat{t_{c}}\left(q,dt\right)$ in each panel is obtained from quantile regressions with 99 values {*q* = 0.01, 0.02, …, 0.99} for the S&P 500 1987 bubble. The red horizontal and vertical dashed lines represent the true critical time *T*_*c*_ = 1987.10.19. The black tilted dashed line represents the diagonal line *t*_*c*_ = *t*_*end*_.

Fig 3 shows the dependence of $\hat{t_{c}}\left(q,dt\right)$ as a function of *t*_*end*_ for two different values of the window size *dt* = 500 and 750 trading days (*dt* = *t*_*end*_ − *t*_*start*_), obtained by quantile regressions with 99 values {*q* = 0.01, 0.02, …, 0.99} of the S&P 500 time series already used in Fig 1 in windows sliding in steps of 5 trading days within [1986.05.12, 1988.08.29]. Their medians (black squares) and averages (red stars) are determined and compared with the *L*^2^ estimates shown as the blue triangles. At the scale *dt* = 500 days in Fig 3A, one can observe that the predicted medians and averages starting from *t*_*end*_ = May 1987 become stable and close to the true critical date *T*_*c*_ = 1987.10.19 (represented by the red dashed horizontal and vertical lines). In contrast, the *L*^2^ estimate is more unstable. At the scale *dt* = 750 days in Fig 3B, a remnant of the stability observed at the scale *dt* = 500 days is visible but the prediction is much more noisy.

The first important message of Fig 3 is that, when *t*_*end*_ is too far from *T*_*c*_, the estimated $\hat{t_{c}}$ is not stable and systematically underestimates the time of the bubble burst. Moreover, $\hat{t_{c}}$ is found to move upward proportionally to *t*_*end*_ as the later increases. This observation holds for all three estimators (i.e., average, median and OLS fit). The second message is that the difference between the averages and medians shows that the distribution of these estimates is non-normal and skewed.

#### Quantile-Violin representation *q*(*t*_*c*_) − *pdf*(*t*_*c*_(*q*)) of the ensemble of quantile regression functions

The results of Fig 3 are far from constituting the whole story since the quantile regressions can give much more than just an average or median tendency. In order to capture the wealth of information of those 99 functions obtained for each *t*_*end*_, we introduce a generalisation of the violin plot [37] and call it “Quantile-Violin plot” (represented by *q*(*t*_*c*_) − *pdf*(*t*_*c*_(*q*))), in which the standard box plot is complemented by a rotated kernel density plot on its right side, and the corresponding *q* values are given on the left side.

**Fig 4:**
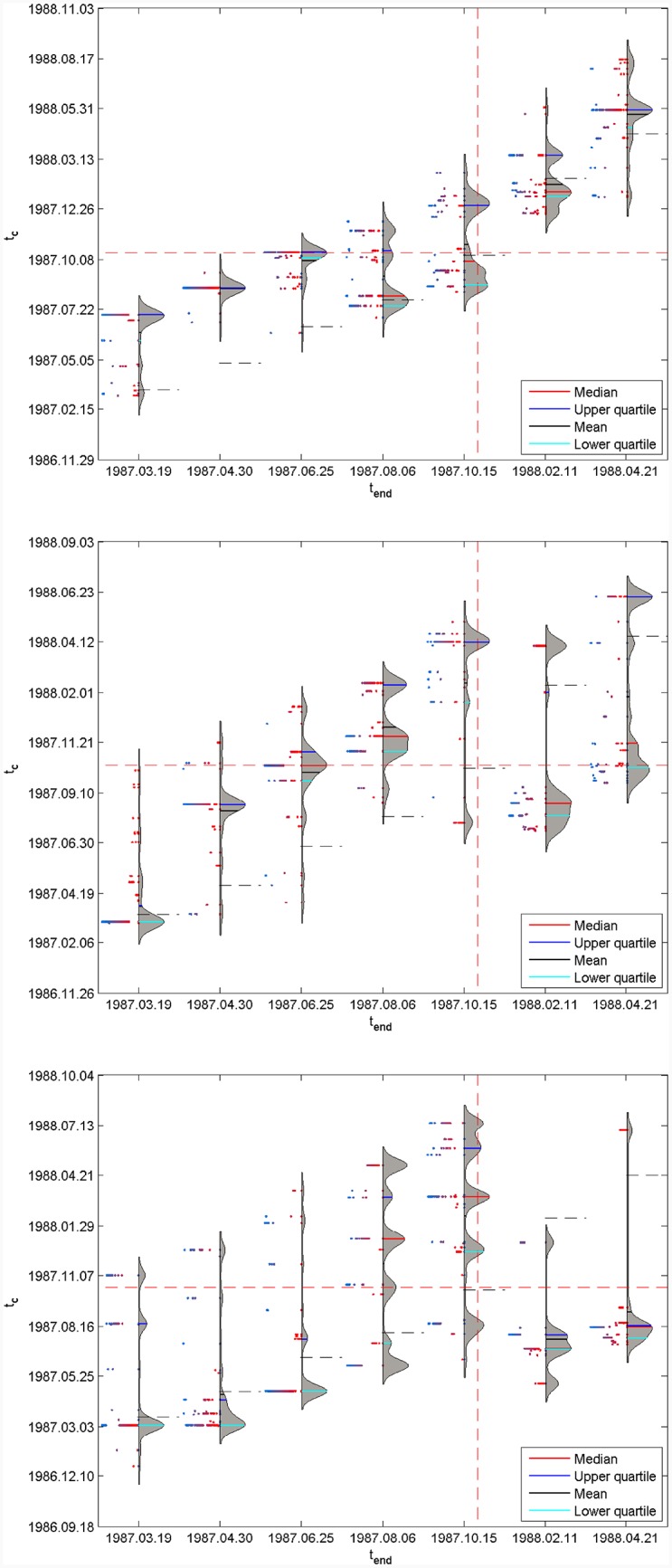
Quantile-Violin plots of $\hat{t_{c}}$ for the seven values of *t*_*end*_. (A) *dt* = 500 trading days. (B) *dt* = 750 trading days. (C) *dt* = 1000 trading days. Each panel shows the results of the analysis for the following set of *t*_*end*_ = 1987.03.19, 1987.04.30, 1987.06.25, 1987.08.06, 1987.10.15, 1988.02.11 and 1988.04.21. $\hat{t_{c}}$ is determined by quantile regression of the log-price of the S&P 500 1987 bubble with Formula (4) with the search space: *m* ∈ [0, 2], *ω* ∈ [1, 50] and *t*_*c*_ ∈ [*t*_*end*_ − 0.20*dt*, *t*_*end*_ + 0.20*dt*]. For each *t*_*end*_, the right side of the Quantile-Violin plot gives the rotated kernel density function of $\hat{t_{c}}$ over the set of 99 quantiles, as well as the descriptive statistics, such as the median (red line), the upper quartile (blue line), the mean (black line) and the lower quartile (brilliant blue line). These values can be read on the scale along the main vertical axis *t*_*c*_ axis. The left side of the Quantile-Violin gives values of *q* for each $\hat{t_{c}}$ contributing to the distribution on the right side, with *q* = 0 on the axis and *q* = 1 corresponding to the maximum extension to the left. The red dashed horizontal and vertical lines represent the real critical date *T*_*c*_ = 1987.10.19.

Specifically, Fig 4 plots the results for the S&P 500 1987 bubble, where the three panels correspond to *dt* = 500, 750 and 1000 trading days, respectively. Each panel contains seven Quantile-Violin plots associated with the seven *t*_*end*_ = 1987.03.19, 1987.04.30, 1987.06.25, 1987.08.06, 1987.10.15, 1988.02.11 and 1988.04.21. For a given *t*_*end*_, the right side of the Quantile-Violin plot gives the rotated kernel density function of $\hat{t_{c}}$ over the set of 99 quantiles, as well as the descriptive statistics, such as the median (red line), the upper quartile (blue line), the mean (black line) and the lower quartile (brilliant blue line). These values can be read on the scale along the main vertical *t*_*c*_ axis. The left side of the Quantile-Violin plot gives values of *q* for each $\hat{t_{c}}$ contributing to the distribution on the right side, with *q* = 0 on the central axis and *q* = 1 corresponding to the maximum extension to the left. The red dashed horizontal and vertical lines represent the real critical date *T*_*c*_ = 1987.10.19.

For *dt* = 500 trading days in Fig 4A, one can observe the stabilisation for *t*_*end*_ = 1987.06.25, 1987.08.06 and 1987.10.15 of a set of scenarios bracketing the true critical time *T*_*c*_ = 1987.10.19. Earlier *t*_*end*_’s predictions are too far from *T*_*c*_ to have it in their prediction horizon. But, there are scenarios in which $\hat{t_{c}}$ tends to be stable and much closer to the true value than the mean, median or OLS estimates. A qualitatively similar picture emerges for *dt* = 750 days in Fig 4B, albeit more murky, with a larger spread of the estimated $\hat{t_{c}}$’s. A similar behavior is obtained for the larger time scale *dt* = 1000 days in Fig 4C, with an even broader set of scenarios around the true *T*_*c*_. When *t*_*end*_ is close to *T*_*c*_, one can also see how sensitive the quantile regressions are as five main scenarios appear corresponding to five modes of the distribution of $\hat{t_{c}}$.

From a statistical point of view, the main message of Fig 4 is that the probability density function of $\hat{t_{c}}$ is multimodal. The Quantile-Violin plots provide a more in-depth view of the unfolding scenarios obtained by the LPPLS quantile regressions performed with the search ranges *m* ∈ [0, 2], *ω* ∈ [1, 50] and *t*_*c*_ ∈ [*t*_*end*_ − 0.20*dt*, *t*_*end*_ + 0.20*dt*]. These Quantile-Violin plots also indicate the primary virtue of the median of quantile estimates [33]: (i) if the true *T*_*c*_ falls within the range encompassed by all forecasts, no more than half of the individual forecasts will be superior to the median forecast; (ii) at worst, if the true *T*_*c*_ lies outside the forecast range, the median forecast will be better than 50% of the forecasts.

**Fig 5:**
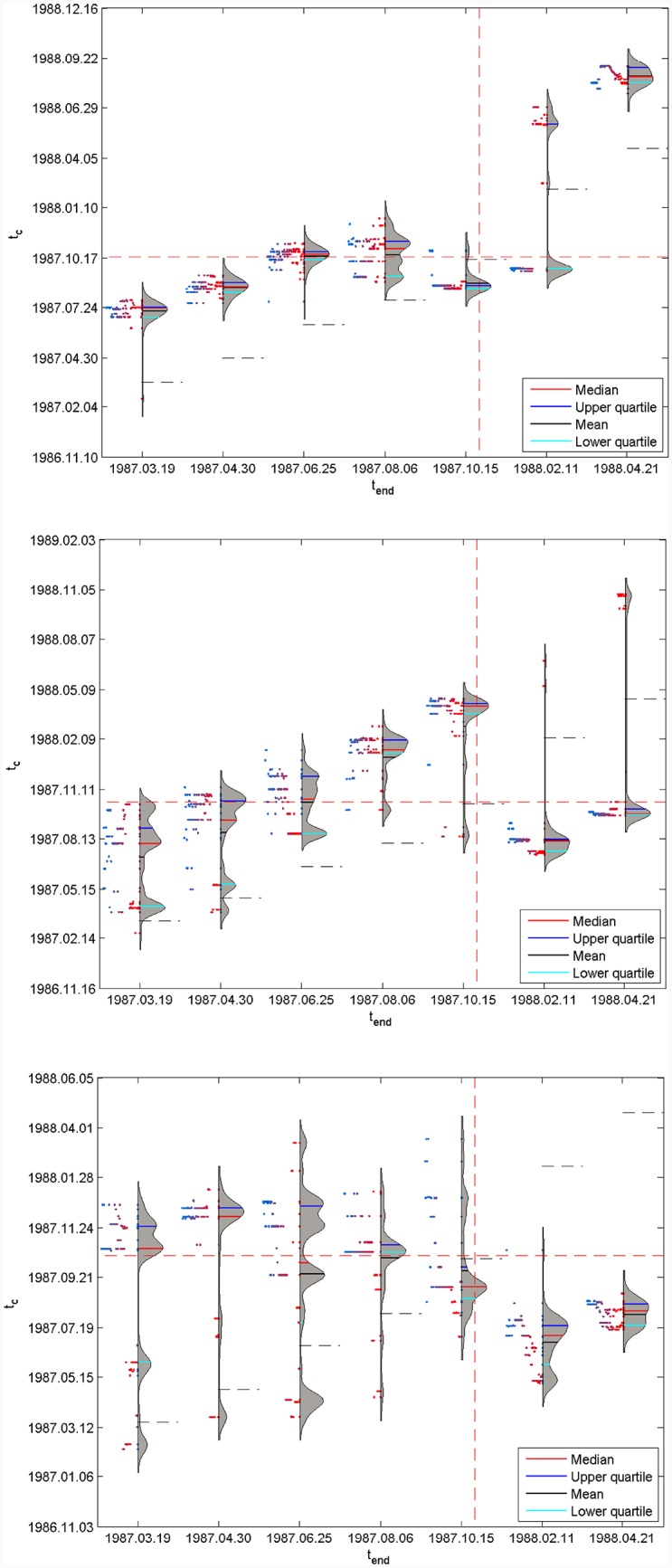
Same as Fig 4 but for the search space *m* ∈ [0.1, 0.9], *ω* ∈ [6, 13] and *t*_*c*_ ∈ [*t*_*end*_ − 0.20*dt*, *t*_*end*_ + 0.20*dt*].

Fig 5 is the same as Fig 4 but for LPPLS quantile regressions performed with the more restrictive search conditions *m* ∈ [0.1, 0.9], *ω* ∈ [6, 13] and *t*_*c*_ ∈ [*t*_*end*_ − 0.20*dt*, *t*_*end*_ + 0.20*dt*], which are derived from previous investigations [5, 35, 36]. Reducing the search space of the two key nonlinear LPPLS parameters *m* and *ω* has two major effects: (i) the distributions of $\hat{t_{c}}$ tend to be more stable as a function of *t*_*end*_ and bracket the true *T*_*c*_ for all cases, except for the earliest *t*_*end*_ = 1987.03.19 at the shortest time scale *dt* = 500 days; (ii) the spreads of $\hat{t_{c}}$ values over the different scenarios are narrower, indicating that the LPPLS quantile regressions provide more precise predictions of the true *T*_*c*_.

#### *dt*-Violin representation *dt*(*t*_*c*_) − *pdf*(*t*_*c*_(*dt*)) of the ensemble of quantile regression functions

**Fig 6:**
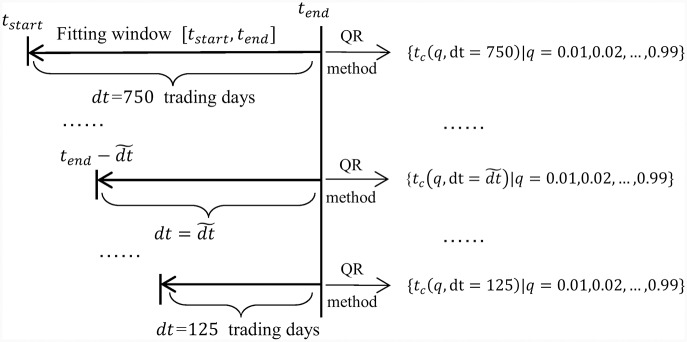
Schematic of the procedure. For each *t*_*end*_, *t*_*start*_ is scanned to give 126 windows of sizes ranging from *dt* = 750 to 125 trading days, in each of which the quantile regression is performed.

Previous works have shown the importance of a multi-scale analysis (see e.g., [38]). In our case, for a fixed *t*_*end*_, this amounts to scan *t*_*start*_ and redo the analysis for each window. Specifically, we shift *t*_*start*_ = *t*_*end*_ − *dt* in steps of 5 trading days, obtaining 126 windows of sizes *dt* = 750, 745, …, 125 trading days. For each window [*t*_*start*_, *t*_*end*_], we perform the OLS estimation and the quantile regression of the model Eq (4) on the same time series already used in Figs 1 and 3–5, obtaining a set $\left\{\hat{t_{c}}\left(q,dt\right)|q=0.01,0.02...0.99\right\}$. This procedure is summarised in Fig 6.

**Fig 7:**
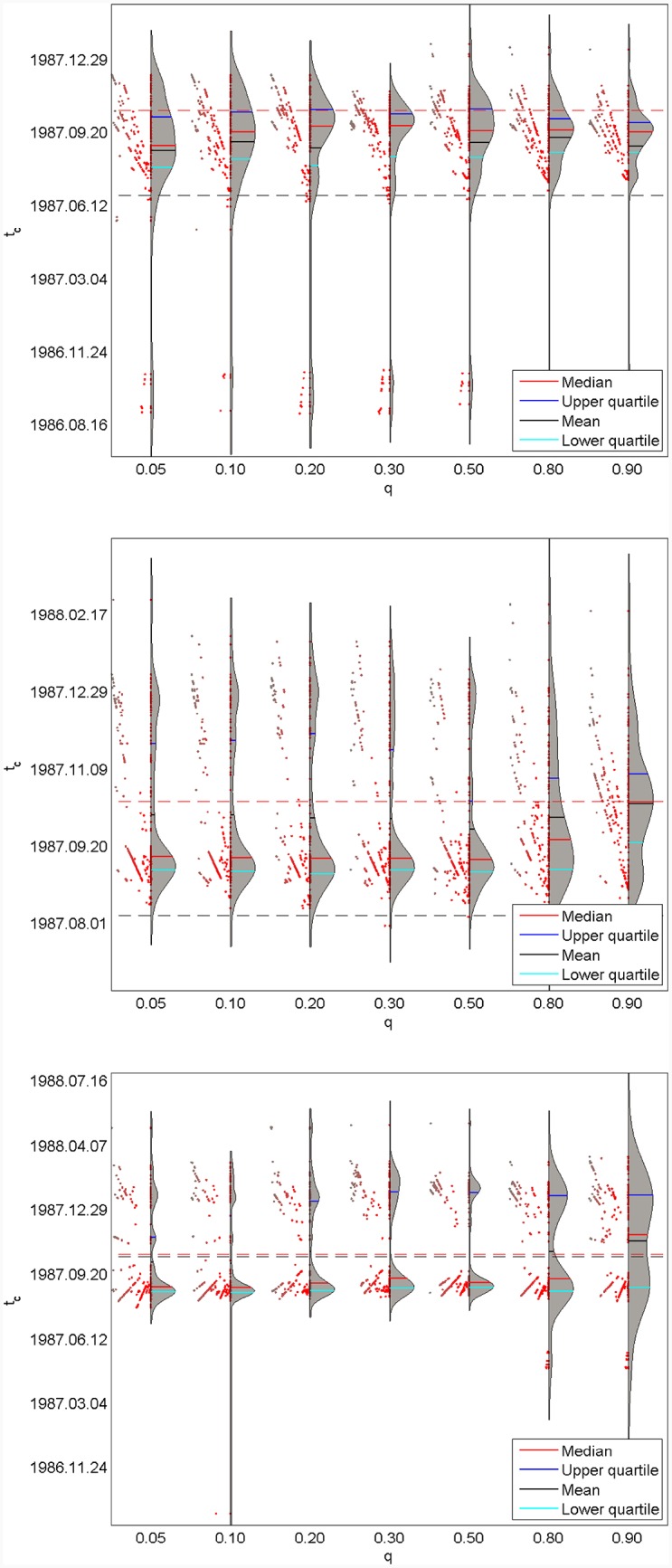
Seven *dt*-Violin plots of $\hat{t_{c}}$ for the S&P 500 1987 bubble. (A) *t*_*end*_ = 1987.06.25. (B) *t*_*end*_ = 1987.08.06. (C) *t*_*end*_ = 1987.10.15. Each panel is associated with the seven values of *q* = 0.05, 0.10, 0.20, 0.30, 0.50, 0.80, 0.90. Each *dt*-Violin plot is constructed over the statistics obtained over the set {*dt* = 750, 745, …, 125 trading days}. The black dashed horizontal lines in each panel indicates *t*_*end*_. The red dashed horizontal line shows the *T*_*c*_ = 1987.10.19.

Analogously to Figs 4, 5 and 7 presents a synopsis of the results concerning the estimation of $\hat{t_{c}}$, but now over the population of the 126 windows for the fixed *t*_*end*_ and the various *q*’s. We further generalise the violin plot [37] in the form of “*dt*-Violin plots”. The standard box plot is now complemented by a rotated kernel density plot of $\hat{t_{c}}$ over the set of 126 windows on its right side for a fixed *q*, and the corresponding *dt* values are added on the left side. Specifically, Fig 7 shows seven *dt*-Violin plots of $\hat{t_{c}}$ for the S&P 500 1987 bubble, where the three panels correspond to *t*_*end*_ = 1987.06.25, 1987.08.06 and 1987.10.15, respectively. Each panel contains seven *dt*-Violin plots associated with the seven values of *q* = 0.05, 0.10, 0.20, 0.30, 0.50, 0.80, 0.90. The kernel density distribution of $\{\hat{t_{c}}\left(dt\right)|dt=750,745,...,125$ trading days} is shown rotated on the right side, as well as some descriptive statistics, such as the median (red line), the upper quartile (blue line), the mean (black line) and the lower quartile (brilliant blue line). The left side of the *dt*-Violin plot gives the values of *dt* for each $\hat{t_{c}}$ contributing to the distribution on the right side. The smallest window size of 125 days is on the central vertical axis of the *dt*-Violin plots while the largest window size of 750 days corresponds to the maximum distance to the left. The black dashed horizontal lines in each panel indicates *t*_*end*_. The red dashed horizontal line shows the *T*_*c*_ = 1987.10.19. This provides an ensemble view of the predicted transition times $\hat{t_{c}}$ over a large set of window scales.

Fig 7A for *t*_*end*_ = 1987.06.25 demonstrates that essentially all *q*’s predictions are approximatively the same, in the sense that the modes of the $pdf(\hat{t_{c}})$ are close to the true critical date *T*_*c*_ = 1987.10.19. When *t*_*end*_ = 1987.08.06 approaches *T*_*c*_ as shown in Fig 7B, the prediction quality deteriorates with the $pdf(\hat{t_{c}})$ both broadening and becoming bimodal. The quantile predictions differ however on the shape of the density distribution of $\hat{t_{c}}$. The quantile fits for *q* = 0.30 to 0.80 have significantly heavier tails towards large values of $\hat{t_{c}}$, even producing a second local mode about three weeks after *T*_*c*_. Only the largest *q* = 0.90 gives a predicted $pdf(\hat{t_{c}})$ with its mode very close to *T*_*c*_. The other quantiles have their main mode earlier, roughly in the middle of *t*_*end*_ and *T*_*c*_. From the perspective of a decision maker, this corresponds to a second possible scenario, which together with the main mode brackets *T*_*c*_. Fig 7C for *t*_*end*_ = 1987.10.15 very close to *T*_*c*_ exhibits a strong bimodal (and a trimodal for the lowest quantiles) structure of the $pdf(\hat{t_{c}})$, bracketing *T*_*c*_ associated with two modes. The main mode occurs about one month and a half earlier than *T*_*c*_ for all *q*, while it is two months later than *T*_*c*_ for the largest *q* = 0.90. As a whole, the left side of each *dt*-Violin plot in the Fig 7 features dots that are organised in rays, showing that the predicted $\hat{t_{c}}$ form several families, and in each family $\hat{t_{c}}$ is an affine function of the size *dt* of the window of analysis.

## Applications to the prediction of the end of four historical bubbles

The previous section has studied the S&P 500 1987 bubble in great details. But this is just one case. We now extend our analysis to three additional historical bubbles listed in Table 1 to explore the ensemble behavior of the prediction of their critical end times over the set $\{\hat{t_{c}}\left(q,dt\right)|dt=750,745,...,125$ trading days} and over 99 quantiles. We refer to these three additional historical bubbles by the names of the involved markets and the years when they burst. The first one is S&P 500 2007, which was studied in [6, 8]. The second and third one are SSEC 2007 and SSEC 2009, discussed in details in [35]. For each bubble, we picked one value of *t*_*end*_, spanning from one to three months before the crash that terminated the bubble at *T*_*c*_, as given in Table 1. And Table 2 gives a list of symbols and their individual descriptions.

**Table 1:** List of four historical bubbles and fixed *t*_*end*_ for analysis.

Asset & Year of crash	Selected *t*_*end*_	*T*_*c*_
S&P 500 1987	1987.08.06	1987.10.19
S&P 500 2007	2007.07.25	2007.10.09
SSEC 2007	2007.09.10	2007.10.18
SZSC 2009	2009.04.23	2009.07.10

**Table 2:** List of symbols.

Symbols	Descriptions
*q*	quantile level
*dt*	time scale, or the duration of the fitting window
*p*(*t*)	asset price as a function of time *t*
*μ*(*t*)	drift (or conditional expected return) as a function of time *t*
*σ*(*t*)	volatility as a function of time *t*
*dW*	increment of a Wiener process (with zero mean and variance equal to *dt*)
*dj*	discontinuous jump such that *j* = 0 before a crash and *j* = 1 after
*κ*	return loss associated with the occurrence of a crash
*h*(*t*)	crash hazard rate as a function of time *t*
*E*_*t*_[⋅]	expectation operator performed at time *t*, conditional on the history up to time *t*
*t*_*c*_	critical time of the end of the bubble
*A*	terminal value of the logarithm of price at *t*_*c*_
*B*	amplitude of the power law acceleration
*C*	amplitude of the log-periodic oscillations
*m*	exponent quantifying the hyperbolic power law describing the super-exponential growth
*ω*	log-periodic angular frequency
λ	scaling ratio of the temporal hierarchy of accelerating oscillations
*ϕ*	phase of the oscillations
*t*_*start*_	starting date of the fitting window
*t*_*end*_	ending date of the fitting window: *t*_*end*_ = *t*_*start*_ + *dt*
*η*	ratio of the search interval for *t*_*c*_
*F*(⋅)	sum of the OLS residuals
*e*_*t*_	residual as a function of time *t*
*S*_*q*_(⋅)	sum of the quantile-dependent residuals
$\hat{\ln p_{q}\left(t\right)}$	calibrated log-price at the quantile probability level *q*
*T*_*c*_	real bubble bursting time
$\hat{t_{c}}$	estimated *t*_*c*_
$pdf(\hat{t_{c}})$	probability density function of $\hat{t_{c}}$

**Fig 8:**
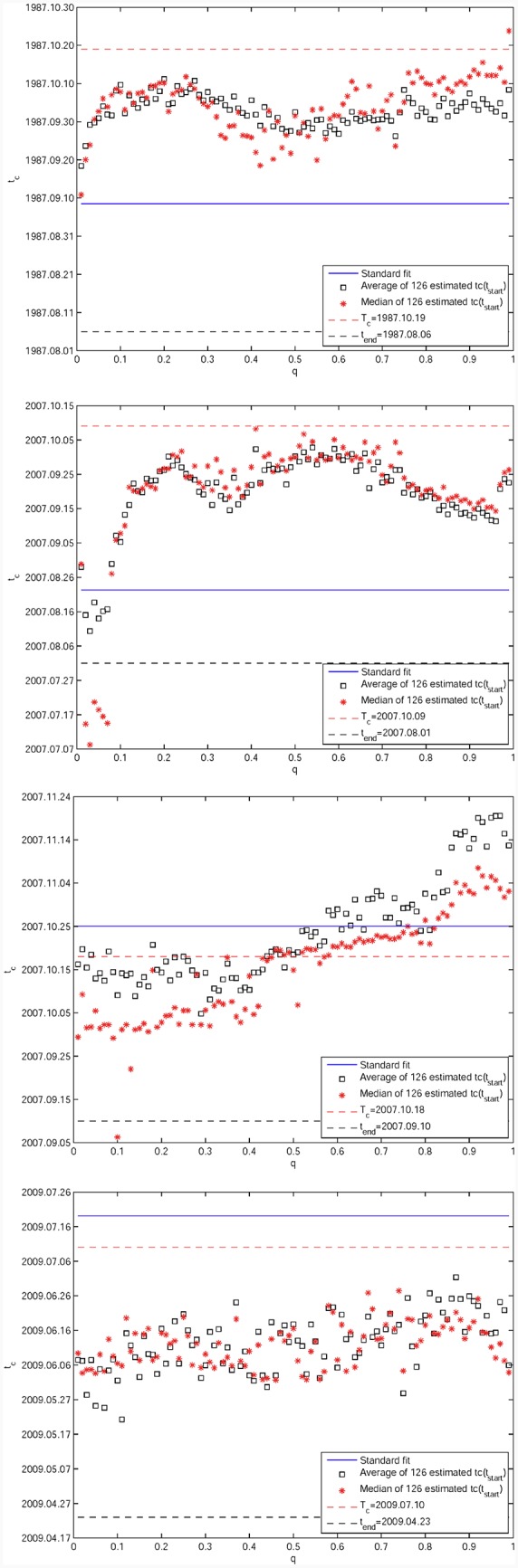
Predicted critical end time $\hat{t_{c}}$ as the function of *q* for the four historical bubbles. (A) S&P 500 1987 bubble. (B) S&P 500 2007 bubble. (C) SSEC 2007 bubble. (D) SZSC 2009 bubble. Each panel shows the medians (red stars) and averages (black squares) of $\hat{t_{c}}$ as functions of *q* = 0.01, 0.02, …, 0.99 for the fixed *t*_*end*_ given in Table 1, over the population of window sizes spanning {*dt* = 750, 745, …, 125}. The blue line is the average of the *L*^2^ calibration based predictions over the same set of window sizes. For each panel, the black dashed line shows the respective position of *t*_*end*_, and the red dashed line shows the corresponding true critical date *T*_*c*_.

Fig 8 shows the medians (red stars) and averages (black squares) of $\hat{t_{c}}\left(q,dt\right)$ as a function of *q* for the fixed *t*_*end*_ given in Table 1, over the population of window sizes spanning {*dt* = 750, 745, …, 125 trading days}. For the S&P 500 1987 bubble in Fig 8A and the S&P 500 2007 bubble in Fig 8B, the results confirm the previous analysis by showing that the LPPLS quantile regressions provide significantly better predictions than the standard *L*^2^ calibration based predictions. Only for the SSEC 2007 bubble in Fig 8C, we observe significant variations of the medians and averages as functions of *q*. For the medians, we see an approximate plateau for *q* between 0.50 and 0.80, which slightly overestimates the true *T*_*c*_ but is earlier than the *L*^2^ calibration based prediction (blue line). Lower (resp. larger) *q*’s predictions underestimate (resp. overestimate) the true *T*_*c*_. In the case of the SZSC 2009 bubble in Fig 8D, all quantiles give again consistent predictions for $\hat{t_{c}}$, which are however too early by about one month. Its *L*^2^ calibration based prediction is closer to the true *T*_*c*_, while slightly overestimating it.

**Fig 9:**
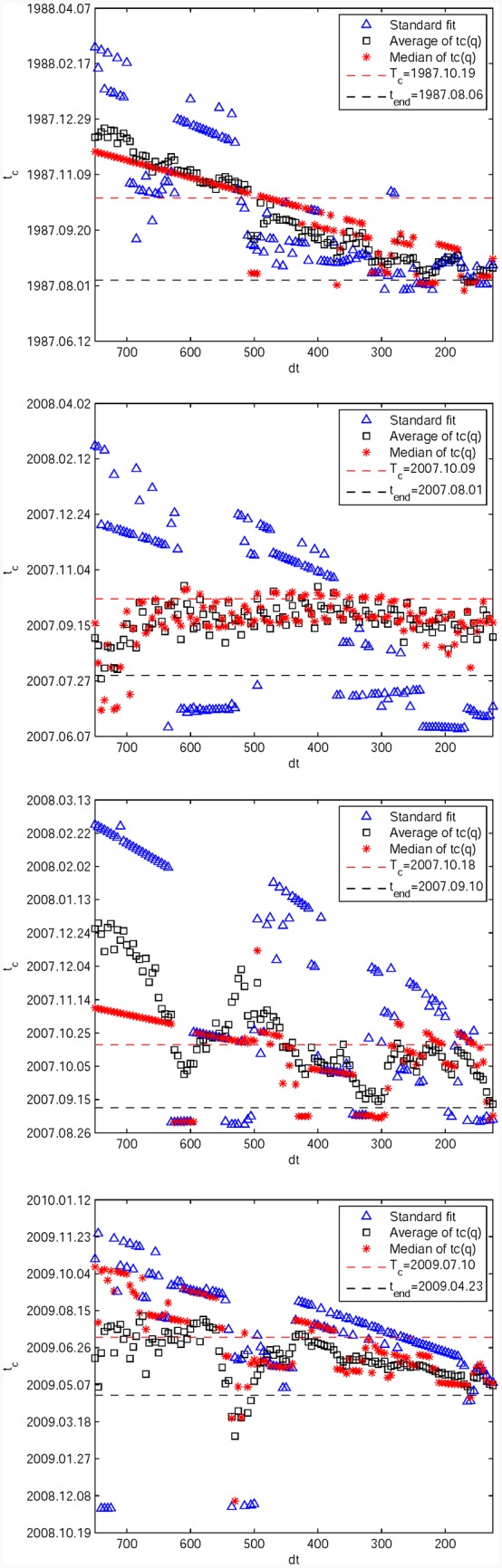
Predicted critical end time $\hat{t_{c}}$ as the function of *dt* for the four historical bubbles. (A) S&P 500 1987 bubble. (B) S&P 500 2007 bubble. (C) SSEC 2007 bubble. (D) SZSC 2009 bubble. Each panel shows the medians (red stars) and averages (black squares) of $\hat{t_{c}}$ as functions of *dt* for the fixed *t*_*end*_ given in Table 1, over the population of *q* values spanning {*q* = 0.01, 0.02, …, 0.99}. The blue triangles are *L*^2^ calibration based predictions for comparation. For each panel, the black dashed line shows the respective position of *t*_*end*_, and the red dashed line shows the corresponding true critical date *T*_*c*_.

Summarising the results of these four cases, the quantile regressions are better than the *L*^2^ calibration in two cases, approximately the same in one case and worse in the last case. For these four bubbles, notwithstanding the multi-ray structure of $\hat{t_{c}}$ as a function of *dt* in Figs 7 and 9 thus shows again the unstable behaviour of *L*^2^ calibrations compared with the LPPLS quantile regressions as a function of the window sizes. For more details, the medians and averages of the 99 *q*’s estimates are shown as functions of {*dt* = 750, 745, …, 125 trading days} for the fixed *t*_*end*_ given in Table 1, over the population of *q* values spanning {*q* = 0.01, 0.02, …, 0.99}. Overall, one can observe a quite erratic behavior of $\hat{t_{c}}$ for the *L*^2^ calibration in the Fig 9, compared to a much more stable behavior for the quantile regressions. The latter exhibit approximate plateaus of stability of the predicted $\hat{t_{c}}$ as a function of *dt*, which gives confidence in the reliability of the detected LPPLS signal as a function of time scale. This is particularly evident for the S&P 500 2007 bubble in Fig 8B, for which the stable plateau extends almost over the whole range of *dt*. In contrast, the standard OLS estimation of $\hat{t_{c}}$ is sensitive to the chosen size *dt* of the window, leading to inconclusive diagnostics. Thus, the quantile regressions introduce stability in the forecasts when they are exploited as an ensemble of scenarios.

## Consolidated DS LPPLS^™^ indicators

The previous sections have presented a wealth of measures, summarised through the use of the Quantile-Violin in Fig 4 and *dt*-Violin plots in Fig 7, which represent the ensemble of predictions for a given present time *t*_*end*_ over the set of quantile levels *q* used in the LPPLS quantile regression, and over the set of time scales (i.e., window sizes) *dt* used in the calibrations. While informative, the effective use of so many fluctuating and often conflicting signals to inform on the danger for a bubble burst and to trigger an actionable decision remains a challenge. To address this, we propose two indicators that aggregate these signals, inspired from previous works on historic bubbles [6, 35, 38] via the implementation of pattern recognition of LPPLS structures and filtering, as suggested in Fig 5. These two indicators have been briefly discussed to present the ex-ante forecast of the Chinese bubble and its burst that started in June 2015 [39].

1. The **DS LPPLS Confidence^™^ indicator** is the fraction of fitting windows whose calibrations meet the filtering condition 1 in Table 3 (within the JLS framework, the condition that the crash hazard rate *h*(*t*) is non-negative by definition [40] translates into the value of Damping larger than or equal to 1). It thus measures the sensitivity of the observed bubble pattern to the 126 time windows of duration from 125 to 750 trading days. A large value indicates that the LPPLS pattern is found at most scales and is thus more reliable. If the value is close to one, the pattern is practically insensitive to the choice of *dt*. A small value of the indicator signals a possible fragility since it is presented in a few fitting windows.
2. The **DS LPPLS Trust^™^ indicator** quantifies the sensitivity of the calibrations to the specific realised instance of the noise in the financial time series. Because the calibration is an attempt to disentangle the LPPLS signal from an unknown realisation of the residuals, we generate bootstrap samples of the original data 100 times and add the residuals to the calibrated LPPLS price that proxy for 100 supposed independent realisations of equivalent price patterns. The DS LPPLS Trust^™^ indicator is defined as the median level over the 126 time windows of the fraction among the 100 synthetic time series that satisfy the filtering condition 2 in Table 3. It thus measures how closely the theoretical LPPLS model matches the empirical price time series, 0 being a bad and 1 being a perfect match.
3. **Arithmetic average** and **geometric average** of the DS LPPLS Confidence^™^ indicator and DS LPPLS Trust^™^ indicator: combining these two indicators is instructive to join the two types of information on the time scale over which the LPPLS signal appears and on the quality of the fits.

**Table 3:** Search space and filtering conditions for the qualification of valid LPPLS fits.

Item	Search space	Filtering condition 1	Filtering condition 2
*m*	[0, 2]	[0.1, 0.9]	[0.1, 0.9]
*ω*	[1, 50]	[6, 13]	[6, 13]
*t*_*c*_	[*t*_*end*_ − 0.2*dt*, *t*_*end*_ + 0.2*dt*]	[*t*_*end*_ − 0.15*dt*, *t*_*end*_ + 0.13*dt*]	[*t*_*end*_ − 0.2*dt*, *t*_*end*_ + 0.12*dt*]
Number of oscillation: $\frac{\omega }{2}\ln\left|\frac{t_{c}-t_{start}}{t_{end}-t_{start}}\right|$	—	[2.5, +∞)	[2.5, +∞)
Damping: $\frac{m|B|}{\omega |C|}$	—	[1.2, +∞)	[1, +∞)
Relative error: $\frac{p_{t}-\hat{p_{t}}}{\hat{p_{t}}}$	—	[0, 0.035]	[0, 0.14]

## Empirical analysis of 16 historical bubbles with the consolidated DS LPPLS^™^ indicators

In order to provide a more extensive test of the LPPLS quantile regression approach, we construct the DS LPPLS Confidence and Trust indicators described in the previous section, for 16 historical bubbles listed in Table 4. These indicators can then be compared with the price time series to allow a judgement of how well they can be associated with bubbles and their terminations. And these bubbles are obtained from the previous studies [1, 5, 39, 41, 42] as well as cases reported at the website of the Financial Crisis Observatory at ETH Zurich (www.er.ethz.ch/financial-crisis-observatory.html). The data was obtained from the Thomson Reuters Datastream.

**Table 4:** List of the 16 historical bubbles.

Asset & Year of crash	Data range	Range of *t*_*end*_
S&P 500 1987	1984.01.02-1987.11.13	1986.11.14-1987.11.13
S&P 500 2007	2004.01.01-2009.12.31	2006.11.15-2009.12.31
DJIA 1929	1926.01.02-1930.12.31	1928.07.07-1930.12.31
Nasdaq Composite Index 2000	1993.01.01-2002.12.31	1995.11.16-2002.12.31
Chile 1991/1994	1987.10.01-2000.12.01	1990.08.15-2000.12.01
Venezuela 1997	1994.01.03-1999.12.30	1996.11.15-1999.12.30
Indonesia 1994/1997	1990.01.03-1999.12.30	1992.11.17-1999.12.30
Malaysia 1994	1991.01.01-1995.12.29	1993.11.15-1995.12.29
Thailand 1994	1990.01.01-1994.12.30	1992.11.13-1994.12.30
Hong Kong 1987/1994/1997	1980.01.02-1999.12.31	1982.11.16-1999.12.31
Hong Kong 2007	2000.01.03-2015.04.10	2003.01.17-2015.04.10
Sugar price	2002.01.01-2013.12.31	2004.11.15-2013.12.31
Brent Oil 2008	1990.01.01-2015.04.16	1992.11.13-2015.04.16
SSEC 2007/2009	2004.01.01-2014.12.31	2006.11.15-2014.12.31
SZSC 2007/2009	2004.01.01-2014.12.31	2006.11.15-2014.12.31
SSEC 2015	2011.02.23-2015.05.12	2014.01.07-2015.05.12

Figs 10–25 present the price time series of the 16 historical bubbles together with the DS LPPLS Confidence and Trust indicators constructed using (i) the *L*^2^ fitting method (green curves) and (ii) the quantile regressions (red curves). Since the Confidence and Trust indicators can be constructed for each quantile level *q*, we choose to present them for their arithmetic over the 9 deciles {*q* = 0.10, 0.20, …, 0.90}. (In detail, for *q* = 0.10 as well as for their arithmetic and geometric averages are shown in S1–S16 Figs).

**Fig 10:**
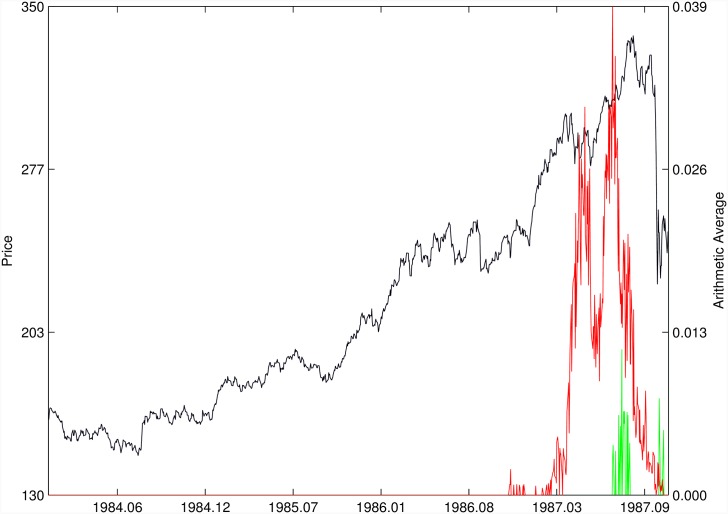
S&P 500 1987 bubble. Arithmetic average of the DS LPPLS Confidence and Trust indicators obtained using quantile regressions with {*q* = 0.10, 0.20, …, 0.90} (red curve) and the standard *L*^2^ calibration method (green curve).

**Fig 11:**
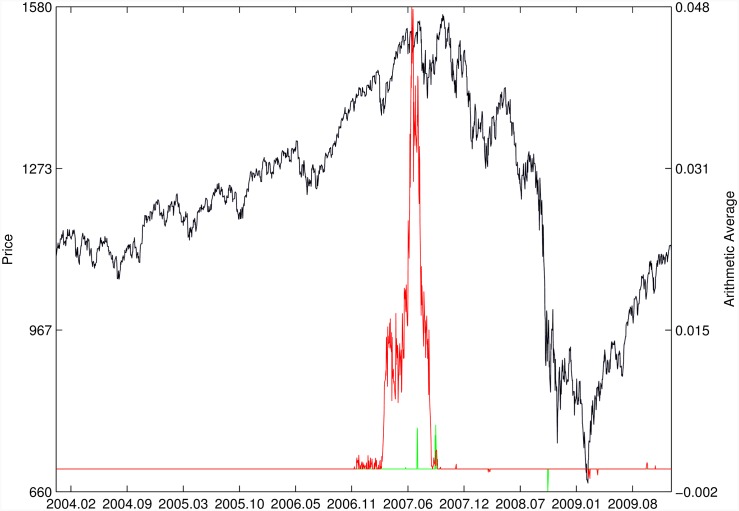
S&P 500 2007. Same as Fig 10.

**Fig 12:**
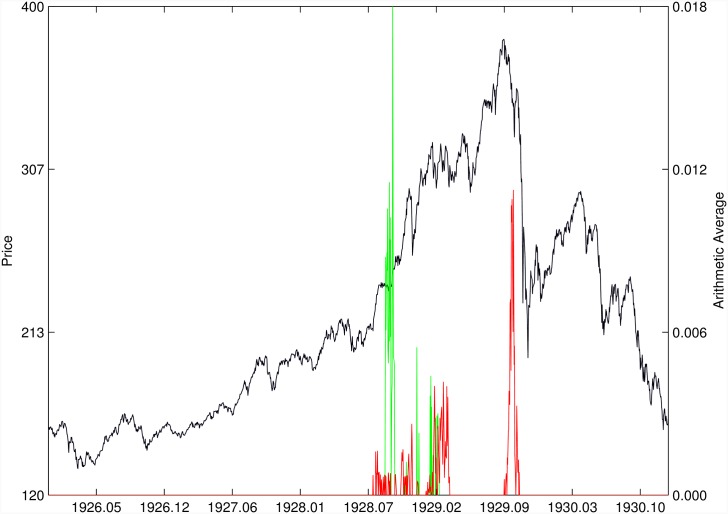
DJIA 1929. Same as Fig 10.

**Fig 13:**
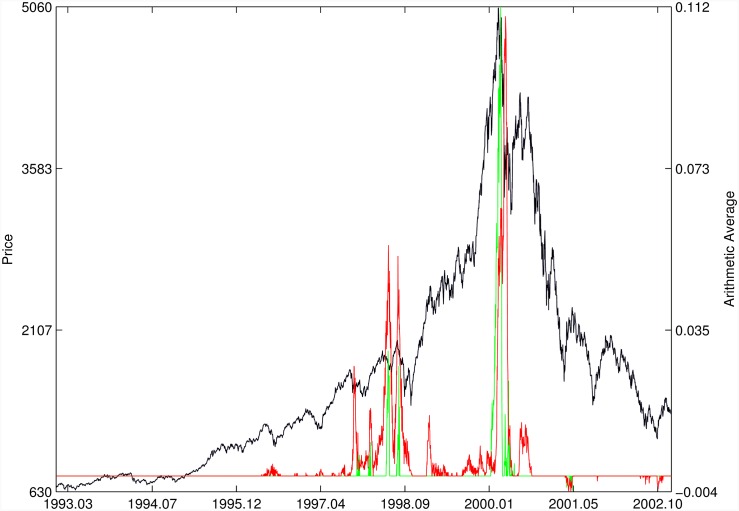
Nasdaq Composite Index 2000. Same as Fig 10.

**Fig 14:**
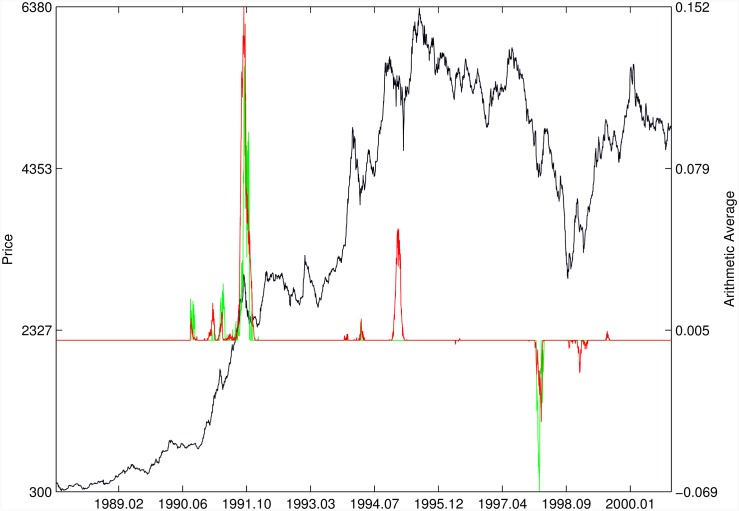
Chile 1991/1994. Same as Fig 10.

**Fig 15:**
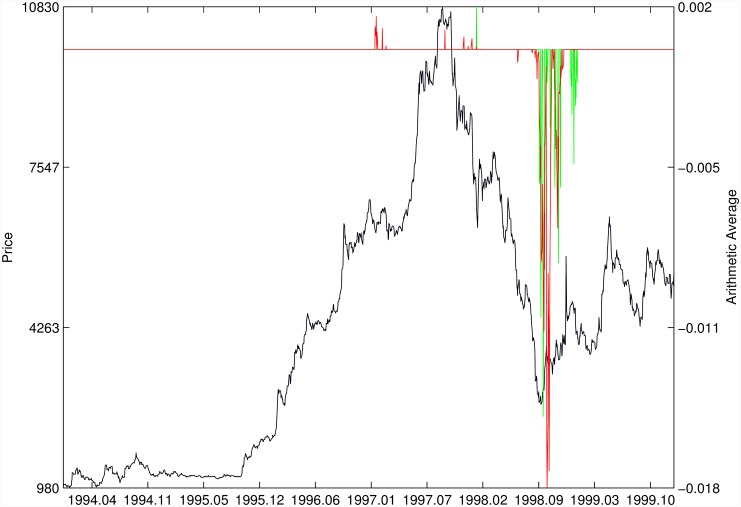
Venezuela 1997. Same as Fig 10.

**Fig 16:**
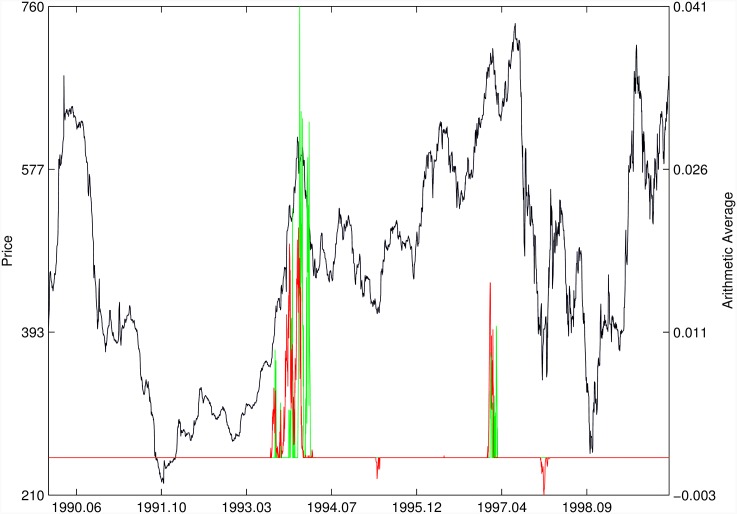
Indonesia 1994/1997. Same as Fig 10.

**Fig 17:**
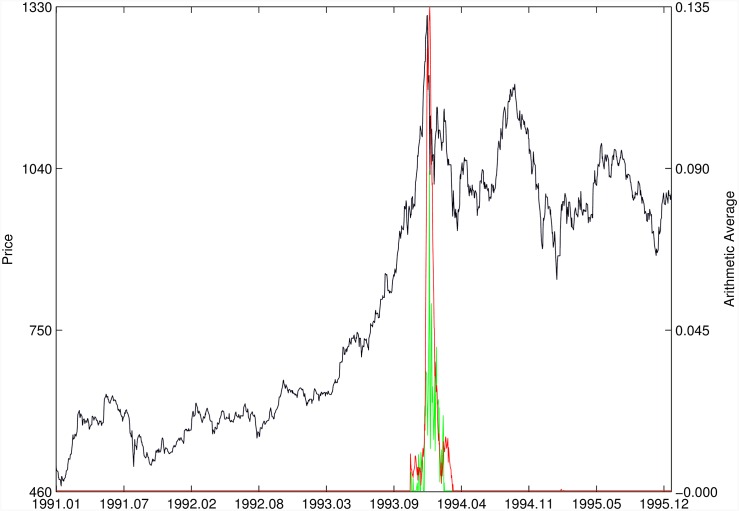
Malaysia 1994. Same as Fig 10.

**Fig 18:**
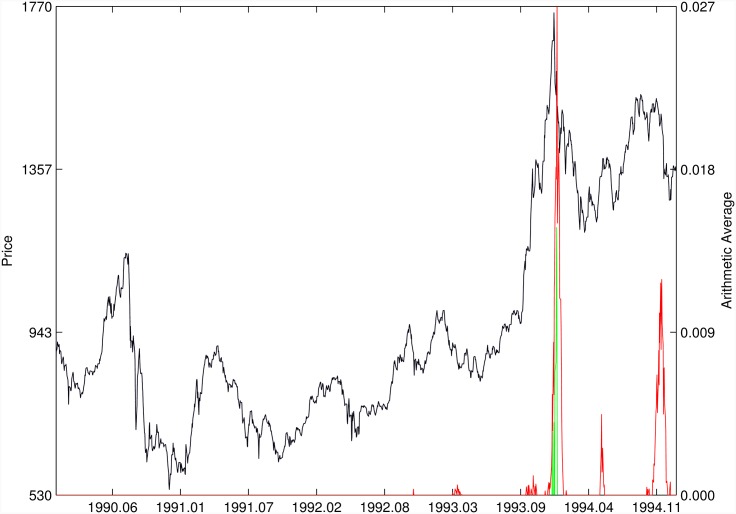
Thailand 1994. Same as Fig 10.

**Fig 19:**
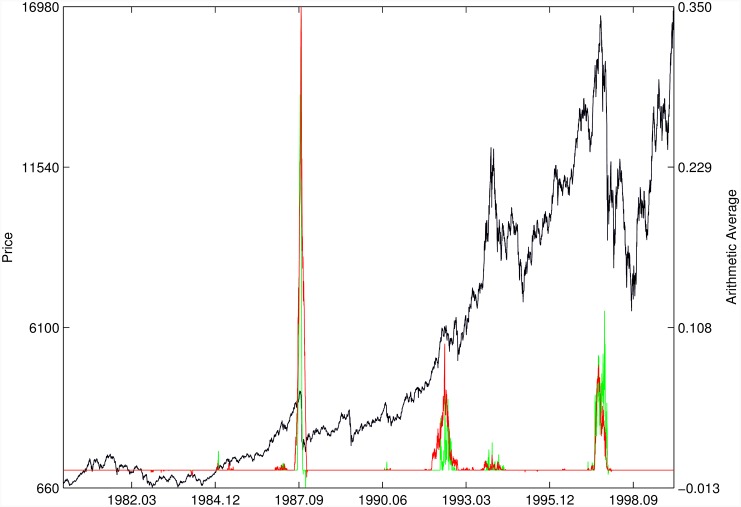
Hong Kong 1987/1994/1997. Same as Fig 10.

**Fig 20:**
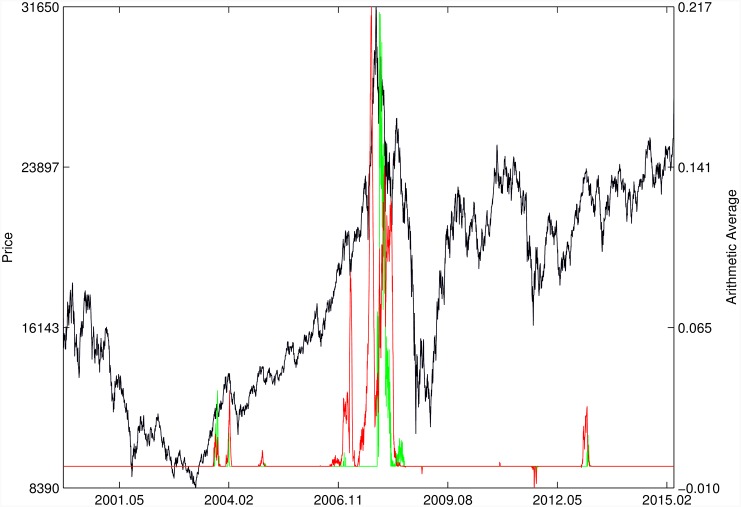
Hong Kong 2007. Same as Fig 10.

**Fig 21:**
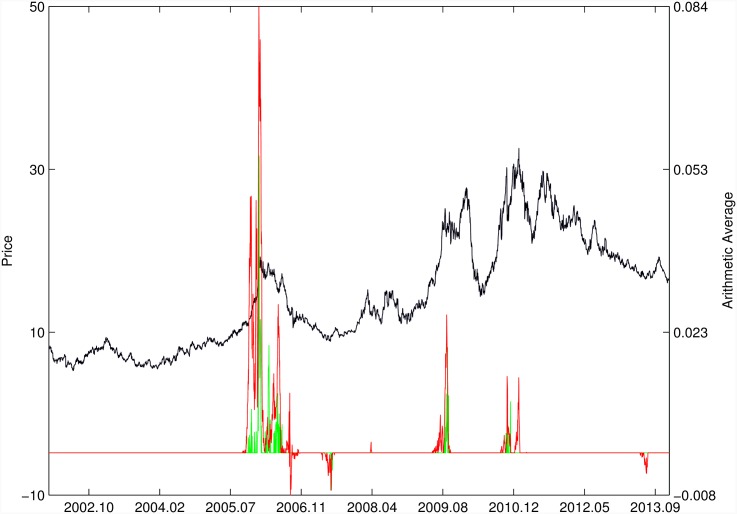
Sugar price. Same as Fig 10.

**Fig 22:**
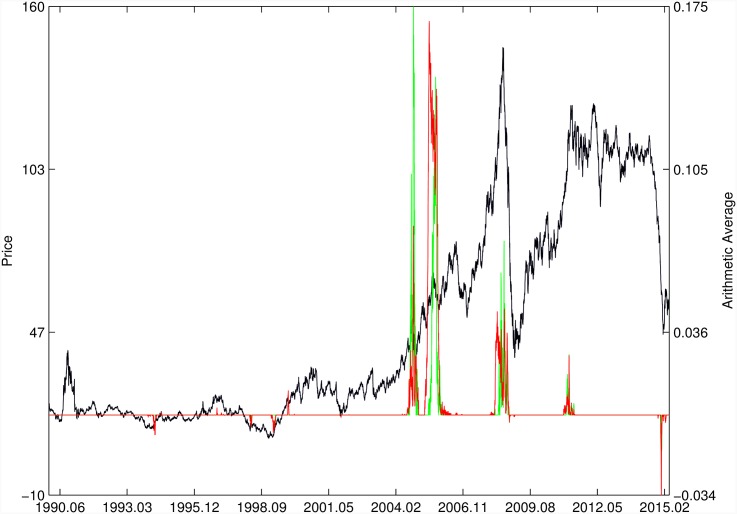
Brent Oil 2008. Same as Fig 10.

**Fig 23:**
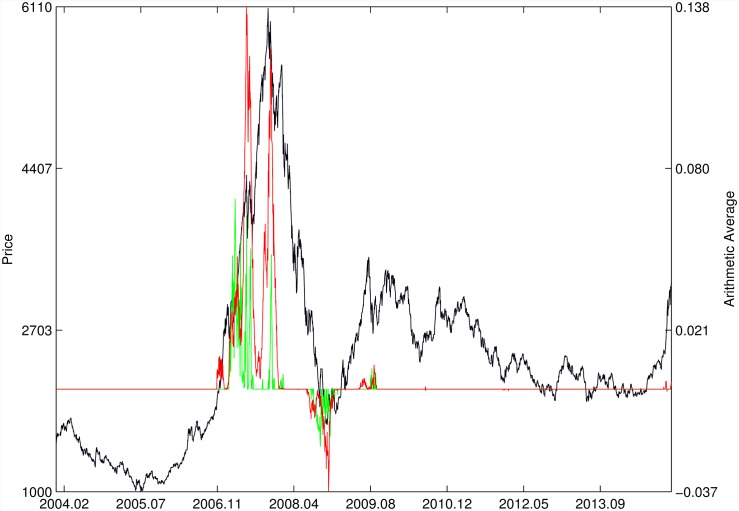
SSEC 2007/2009. Same as Fig 10.

**Fig 24:**
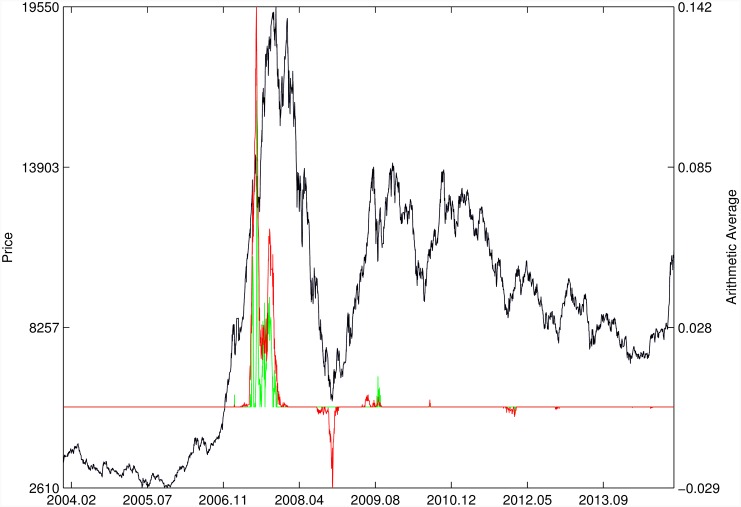
SZEC 2007/2009. Same as Fig 23.

**Fig 25:**
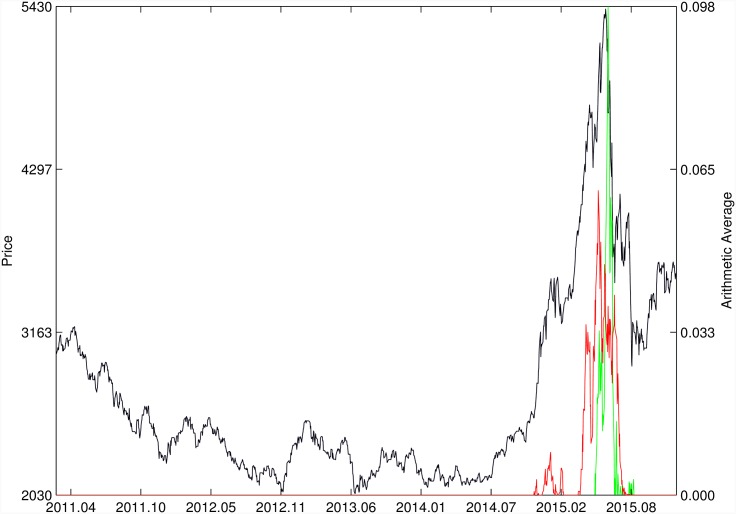
SSEC 2015. Same as Fig 10.

For the S&P 500 1987 bubble in Fig 10 and the S&P 500 2007 bubble in Fig 11, one can observe that the quantile regressions add to the *L*^2^ fitting method by providing in general earlier warning signals, in particular using the lower quantiles *q* = 0.10 in S1 Fig.

For the DJIA 1929 bubble in Fig 12, the quantile regressions provide a neat warning right on target, i.e. just before the crash. Such warning is absent in the *L*^2^ fitting method.

For the Nasdaq Composite Index 2000 bubble shown in Fig 13, the performances of the quantile regression and the *L*^2^ fitting method are similar (a detailed account of the dot-com bubble that crashed in 2000 can be found in Ref. [41]).

For the Chile 1991 and 1994 bubbles shown in Fig 14, one can observe negative values of the indicators that diagnose “negative” bubbles [6, 13], whose end corresponds to a “negative crash” (i.e., a rally or rebound). One can observe that the quantile regressions provide two additional important warning (end of bullish regime in 1994 and rebound in 1998) that are missing in the standard OLS method.

Fig 15 presents the identification of a strong negative bubble and its rebound for the Venezuela 1997 bubble, both by the *L*^2^ fitting method and the quantile regression method that perform similarly. However, the latter provides early warnings of the end of the large preceeding peak, which are absent in the *L*^2^ fitting method.

For the Indonesia 1994/1997 bubble shown in Fig 16, the positive bubbles followed by crashes in 1994 and 1997 are correctly identified by both methods. But again, the quantile regressions provide two negative bubble signals that correctly pinpoint rebounds, which are missed by the *L*^2^ fitting method.

The Malaysia 1994 bubble shown in Fig 17 exhibits a remarkably clean LPPLS pattern, so that all indicators target precisely the peak and subsequent burst. We observe the same joint performance for the Thailand 1994 bubble shown in Fig 18. However, the quantile regressions provide warnings of a large secondary peak after the burst of the first large bubble, which is missed by the *L*^2^ fitting method.

For the Hong Kong market shown in Figs 19 and 20 (see Ref. [7] for a discussion of the set of bubbles and crashes that have punctuated this market again and again), we observe that the *L*^2^ fitting method and quantile regressions provide similar indicators. The same conclusion applies to the price time series of sugar shown in Fig 21, to the Brent Oil bubbles (see Ref. [43] for the analysis of the 2008 bubble) shown in Fig 22 and to the SSEC Chinese index shown in Fig 23 (see Ref. [35] for an early account).

For the SZEC Chinese market shown in Fig 24, the quantile regressions over-perform the *L*^2^ fitting method by identifying precisely the large rebound that occurred in the third quarter of 2008, while the *L*^2^ fitting method completely misses it. Concerning the SSEC 2015 bubble shown in Fig 25, the main difference between the indicators provided by the quantile regressions compared with the *L*^2^ fitting method is that the former provides earlier warnings of the peak of the bubble that occurred in June 2015 as well as signatures of a previous large peak and correction in early 2015. We refer to Ref. [39] for a description of the real-time analysis of the development of the indicators that were used to predict the burst.

Overall, the DS LPPLS Confidence and Trust indicators are found to have strong diagnostic power to identify the market regimes during which prices tend to accelerate upward (resp. downward) and which are followed by strong corrections (resp. rallies). This conclusion holds both for the *L*^2^ fitting method and the quantile regressions. In addition, one can observe a larger sensitivity of the quantile regressions for the detection of negative bubbles and the subsequent rebounds.

## Concluding remarks

This study has shown that positive (resp. negative) bubbles followed by large crashes/corrections (resp. rallies) can be identified by diagnosing the existence of log-periodic power law singular (LPPLS) structures in the log-price dynamics. Given the stochastic nature of log-prices, significant variability in the estimatations and in the predictions is unavoidable. The analysis of their stability and sensitivity with respect to *t*_*end*_, *q* and time scale *dt* is very helpful. We have provided evidence that financial markets exhibit a degree of inefficiency and a potential for predictability, especially during regimes when bubbles develop.

The innovation of the present article includes: (1) the introduction of the quantile regression applied to the LPPLS detection problem, and the comparison with the *L*^2^-based calibration method; (2) the combination of the many quantile regressions with a multi-scale analysis and presentations of the Quantile-Violin and *dt*-Violin plots; (3) the implementation of the DS LPPLS Confidence and Trust indicators through resampling and filtering that finally provides an aggregation and consolidation of the wealth of signals generated at multi-scales and many quantile levels; (4) the detailed analysis of the S&P 500 1987 bubble and the application of the methodology to a total of 16 empirical financial time series exhibiting each at least one massive bubble.

These innovations have the ultimate goal of becoming part of an early warning system that could be run by a central bank, say, to inform it towards appropriate counter measures of impending critical transitions [16, 44–48]. Although the next step of constructing an explicit early warning system is not investigated here [49], the introduction of our new metrics and methodology to develop real world scenarios could provide useful precursors to incorporate in an early warning system.

Overall, the results demonstrate that the quantile regression of LPPLS signals contributes useful early warning signals and the systemic indicators exhibit significant predictive ability around the real critical time when the burst/rally occurs. We also found that the quantile regression method improves on the *L*^2^ based calibration method by providing richer and more stable scenarios. Quantile regression especially focuses on estimating multiple super-exponential rates of change in the quantiles of the distributions of log-price conditional at *t*_*end*_ with different time scales *dt*. It thus presents many new possibilities for the statistical analysis and interpretation of observational data. With the implementation of the systematic indicators, the hybrid form of ensemble forecasting provides a new benchmark on early warning signals of financial crises. From a broader scientific and societal perspective, our article supports a reorientation toward ensemble forecasts based on extracting multi-dimensional information from the noisy signal at multiple scales.

## Supporting Information

S1 Fig S&P 500 1987 bubble. (A) Three groups of DS LPPLS Trust indicator. (B) Three groups of DS LPPLS Confidence indicator. (C) Three groups of the product of DS LPPLS Trust and Confidence indicator. For all panels, the green line is obtained by using the standard *L*^2^ calibration method while the red lines are obtained using quantile regressions. In each panel, the top group is obtained using the first decile *q* = 0.10 quantile regression, the middle group is the arithmetic average over the 9 deciles {*q* = 0.10, 0.20, …, 0.90} and the bottom group is the geometric average over the same 9 deciles {*q* = 0.10, 0.20, …, 0.90}.
(TIF)

S2 Fig S&P 500 2007. Same as S1 Fig.
(TIF)

S3 Fig DJIA 1929. Same as S1 Fig.
(TIF)

S4 Fig Nasdaq Composite Index 2000. Same as S1 Fig.
(TIF)

S5 Fig Chile 1991/1994. Same as S1 Fig.
(TIF)

S6 Fig Venezuela 1997. Same as S1 Fig.
(TIF)

S7 Fig Indonesia 1994/1997. Same as S1 Fig.
(TIF)

S8 Fig Malaysia 1994. Same as S1 Fig.
(TIF)

S9 Fig Thailand 1994. Same as S1 Fig.
(TIF)

S10 Fig Hong Kong 1987/1994/1997. Same as S1 Fig.
(TIF)

S11 Fig Hong Kong 2007. Same as S1 Fig.
(TIF)

S12 Fig Sugar price. Same as S1 Fig.
(TIF)

S13 Fig Brent Oil 2008. Same as S1 Fig.
(TIF)

S14 Fig SSEC 2007/2009. Same as S1 Fig.
(TIF)

S15 Fig SZEC 2007/2009. Same as S1 Fig.
(TIF)

S16 Fig SSEC 2015. Same as S1 Fig.
(TIF)
