# Supplementary figures and images for: Early Warning Signals of Financial Crises with Multi-Scale Quantile Regressions of Log-Periodic Power Law Singularities
Source: PLoS One. 2016 Nov 2;11(11):e0165819. doi: 10.1371/journal.pone.0165819 (PMC5091919; doi:10.1371/journal.pone.0165819)

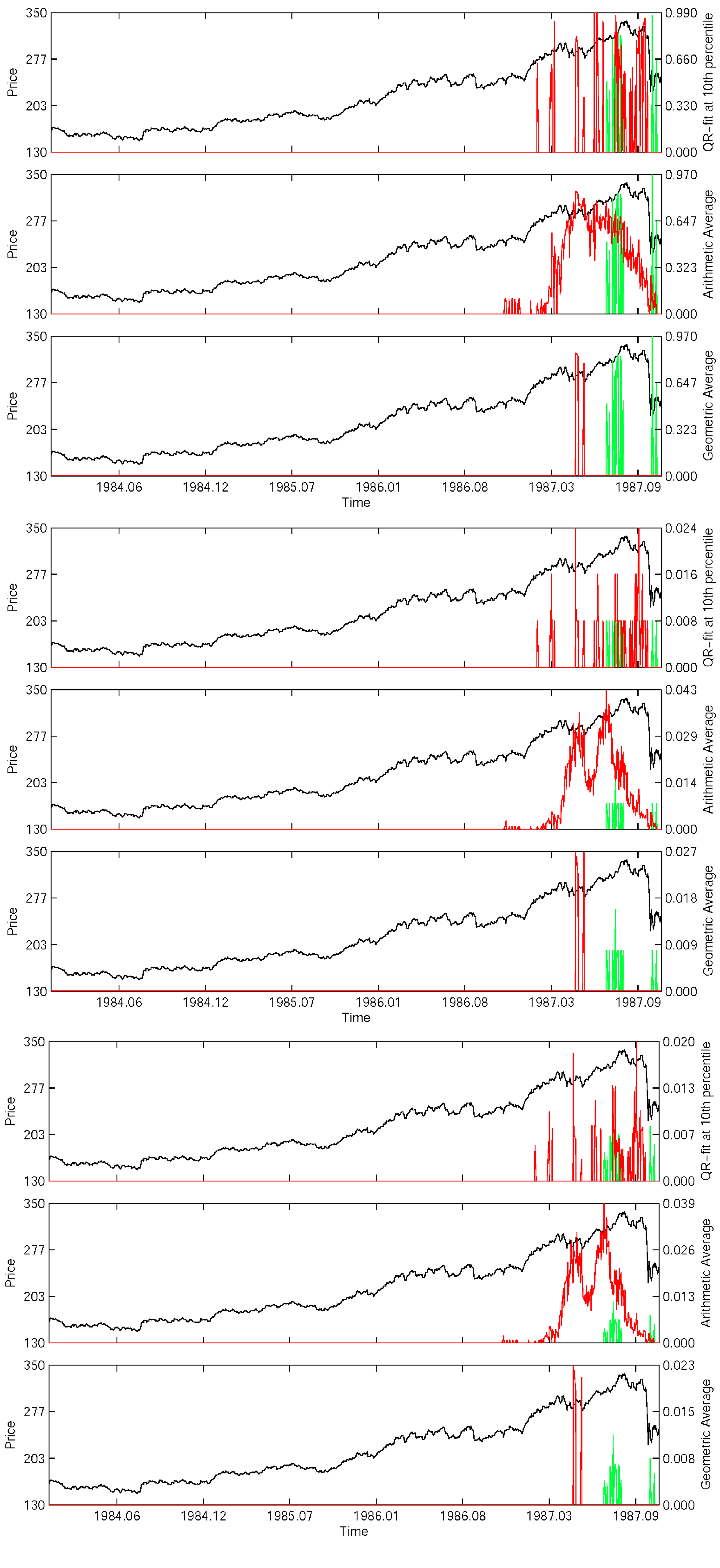

Supplement: S1 Fig — (A) Three groups of DS LPPLS Trust indicator. (B) Three groups of DS LPPLS Confidence indicator. (C) Three groups of the product of DS LPPLS Trust and Confidence indicator. For all panels, the green line is obtained by using the standard L2 calibration method while the red lines are obtained using quantile regressions. In each panel, the top group is obtained using the first decile q = 0.10 quantile regression, the middle group is the arithmetic average over the 9 deciles {q = 0.10, 0.20, …, 0.90} and the bottom group is the geometric average over the same 9 deciles {q = 0.10, 0.20, …, 0.90}. (TIF) [file pone.0165819.s001.tif]

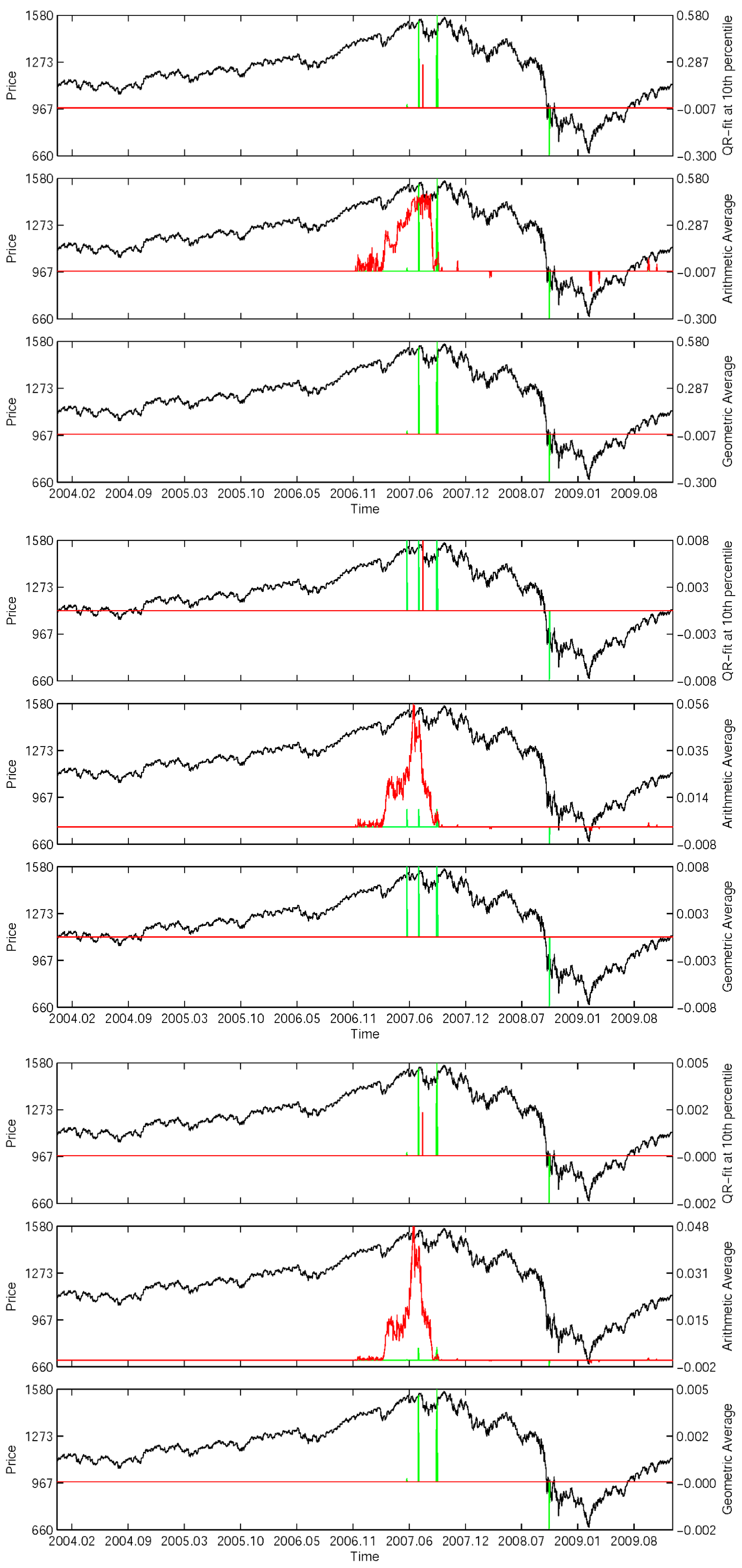

Supplement: S2 Fig — Same as S1 Fig. (TIF) [file pone.0165819.s002.tif]

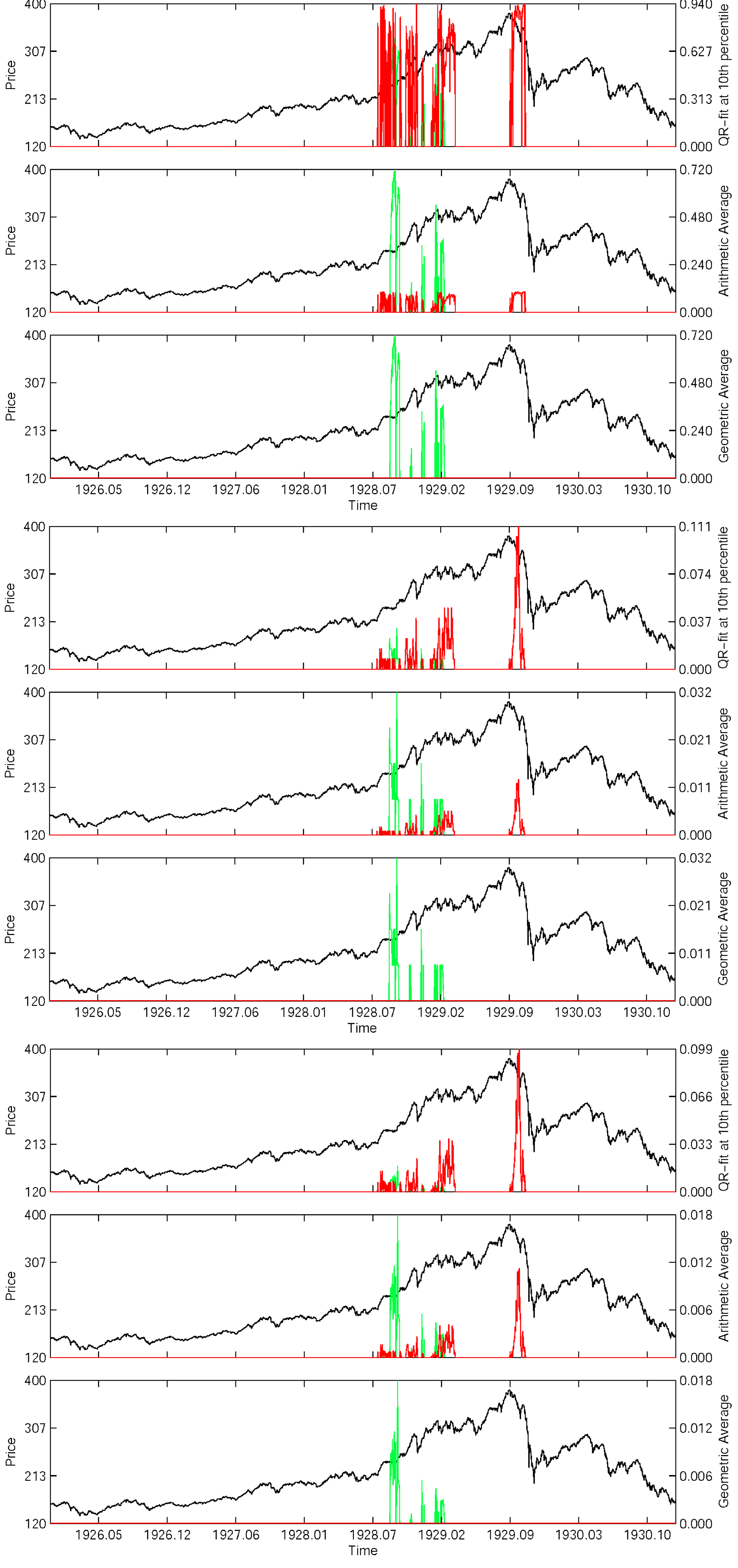

Supplement: S3 Fig — Same as S1 Fig. (TIF) [file pone.0165819.s003.tif]

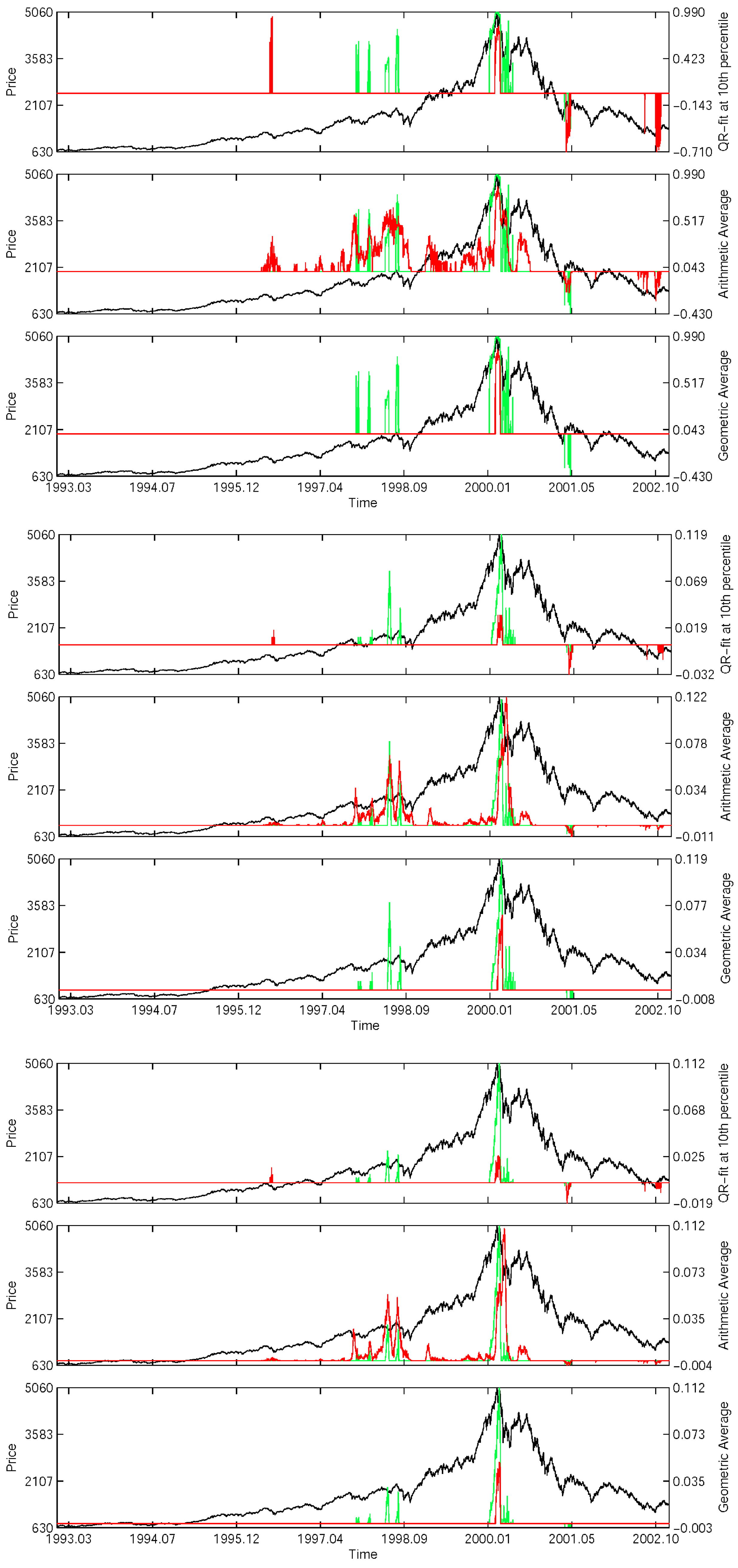

Supplement: S4 Fig — Same as S1 Fig. (TIF) [file pone.0165819.s004.tif]

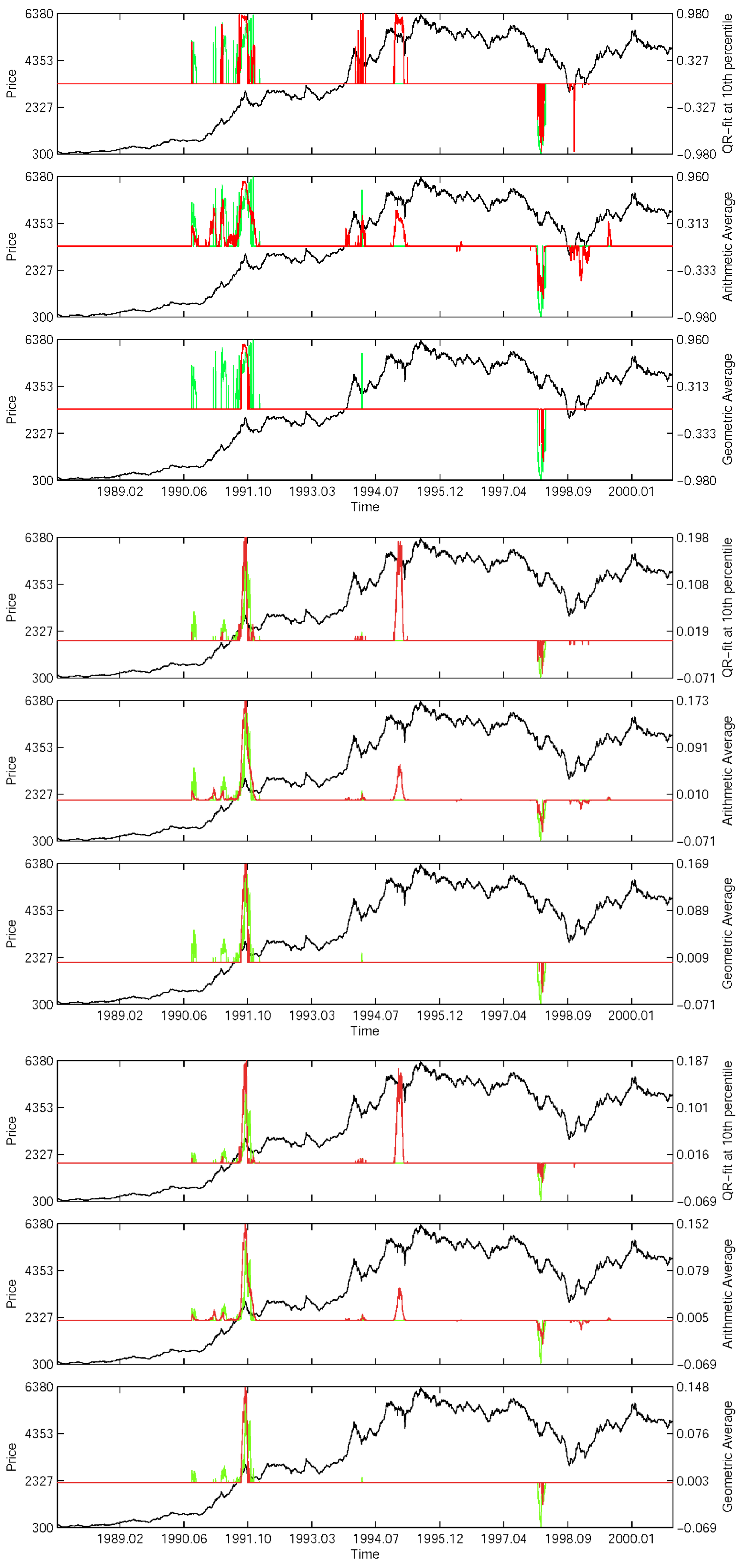

Supplement: S5 Fig — Same as S1 Fig. (TIF) [file pone.0165819.s005.tif]

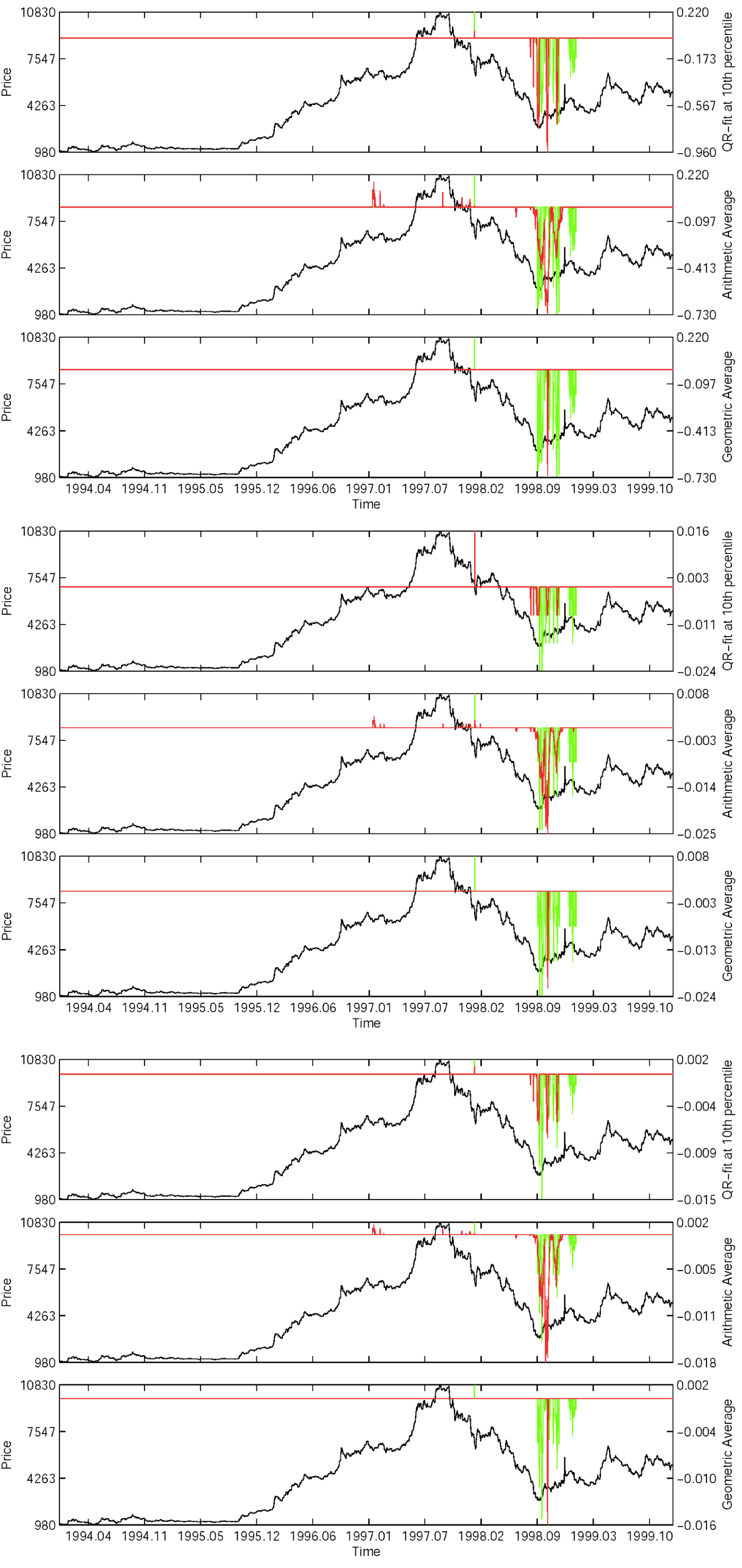

Supplement: S6 Fig — Same as S1 Fig. (TIF) [file pone.0165819.s006.tif]

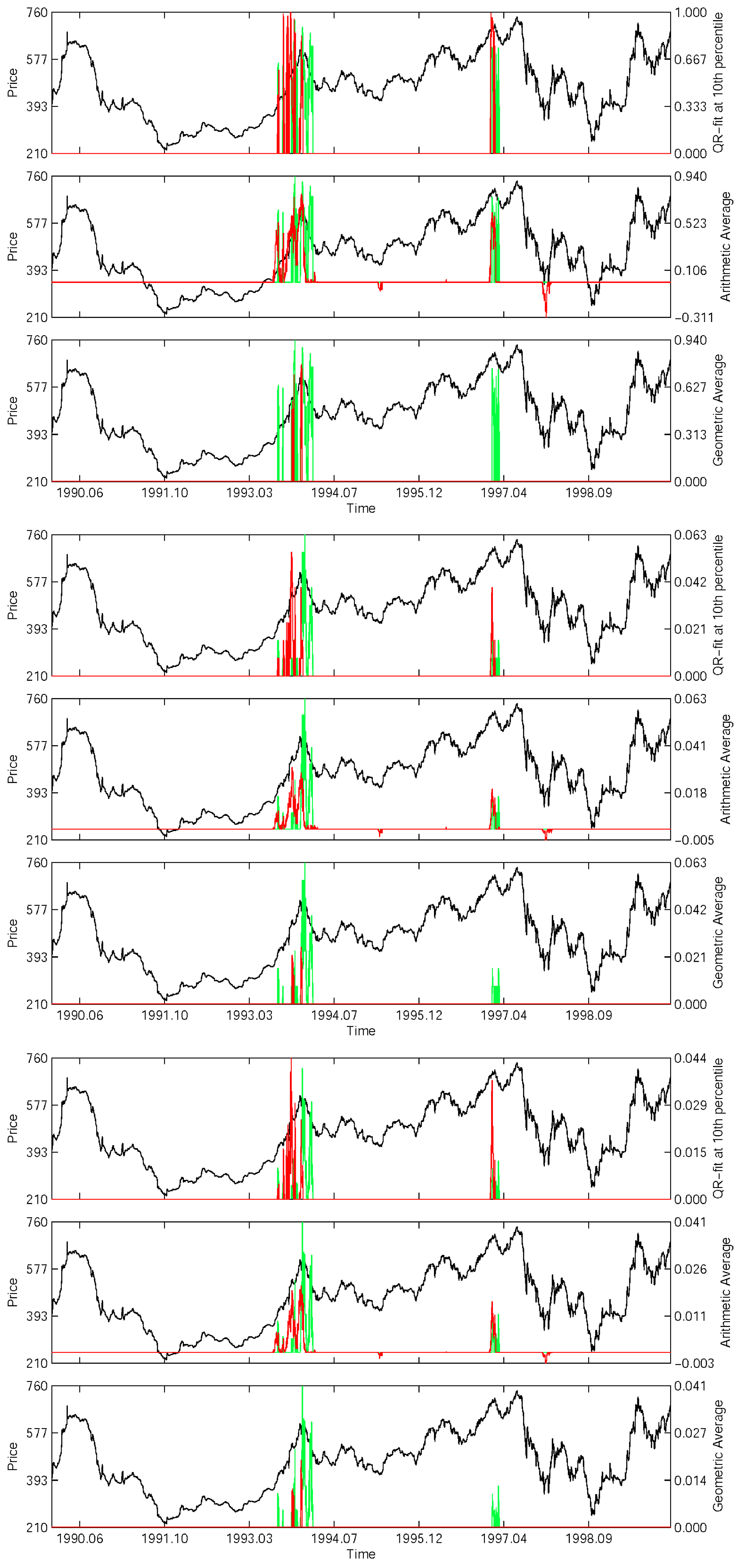

Supplement: S7 Fig — Same as S1 Fig. (TIF) [file pone.0165819.s007.tif]

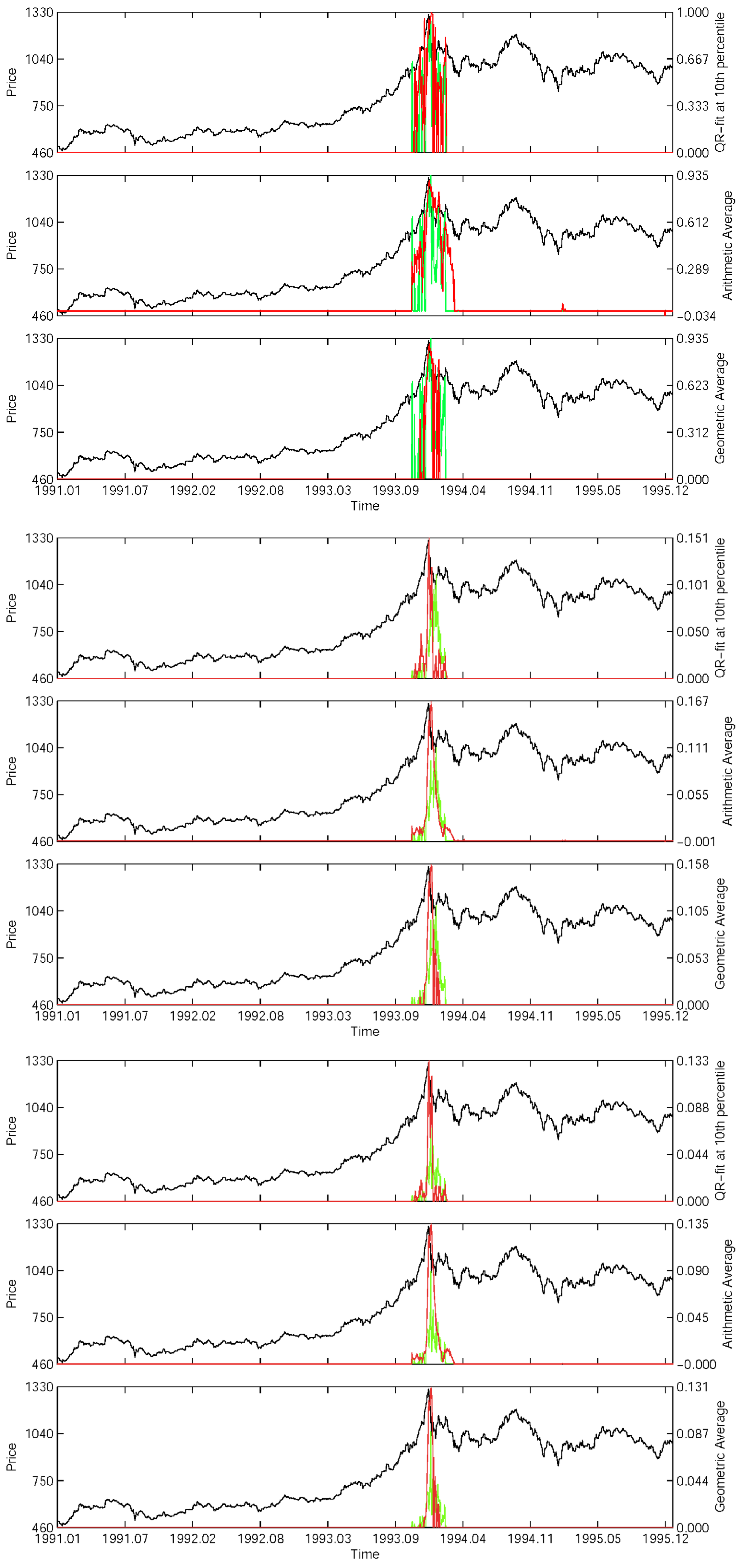

Supplement: S8 Fig — Same as S1 Fig. (TIF) [file pone.0165819.s008.tif]

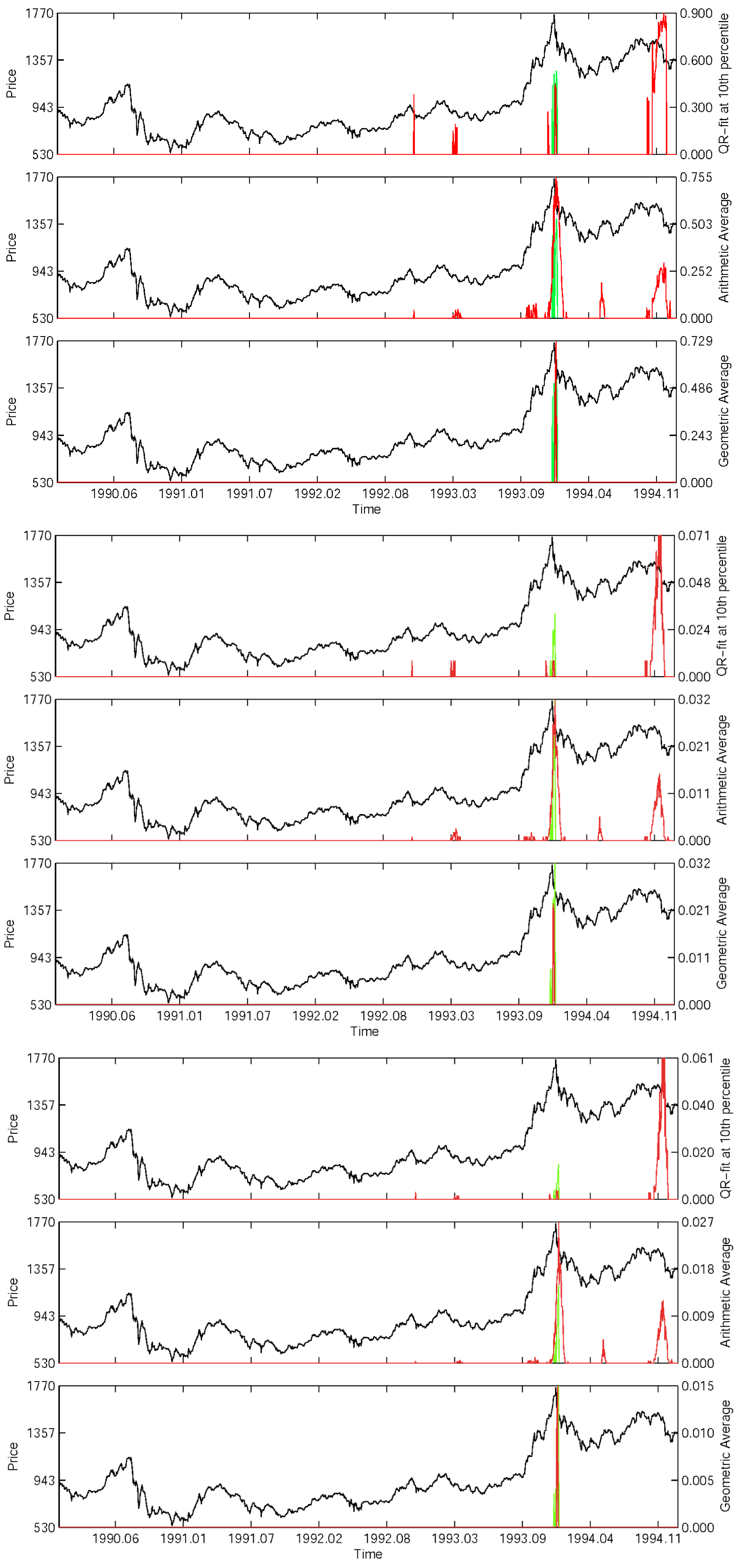

Supplement: S9 Fig — Same as S1 Fig. (TIF) [file pone.0165819.s009.tif]

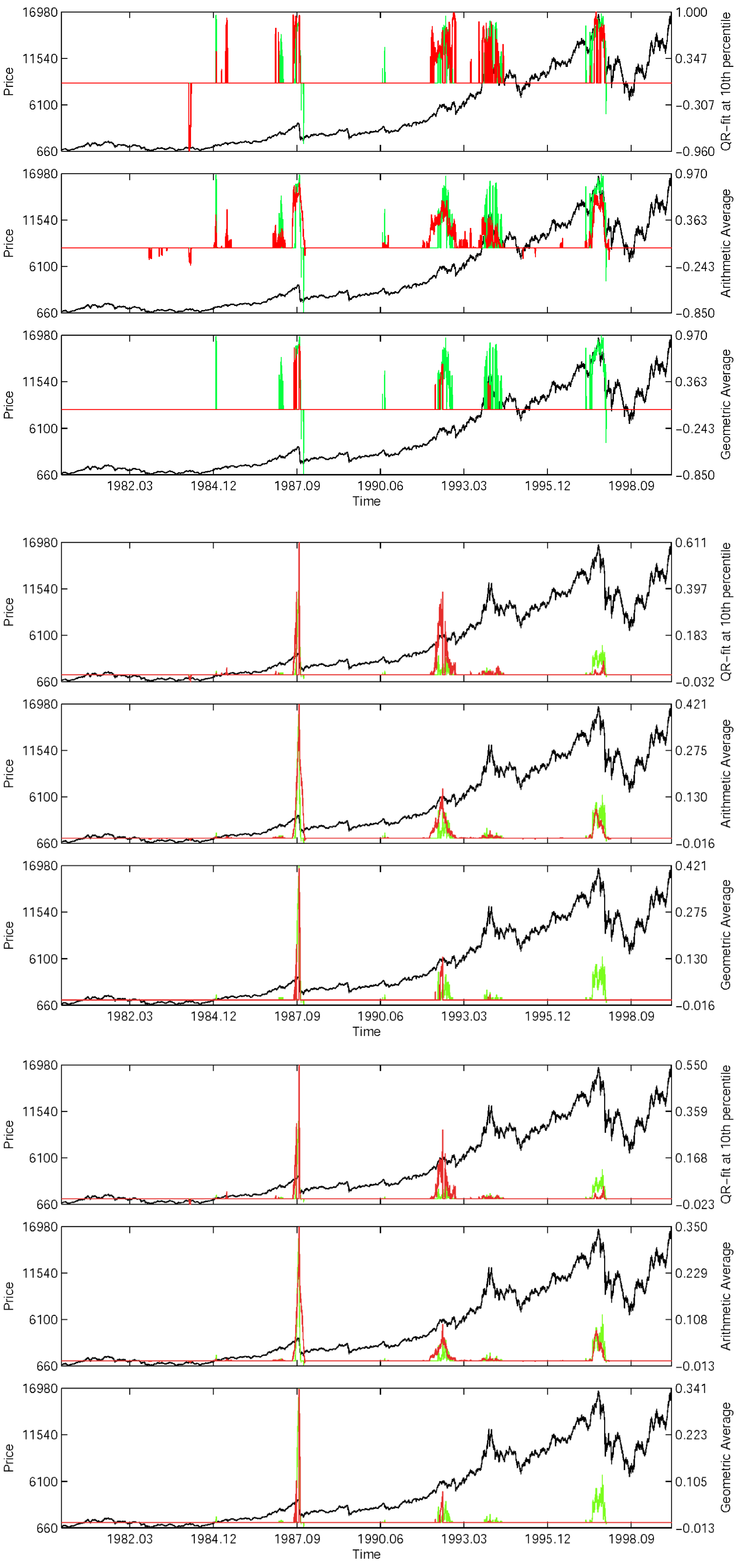

Supplement: S10 Fig — Same as S1 Fig. (TIF) [file pone.0165819.s010.tif]

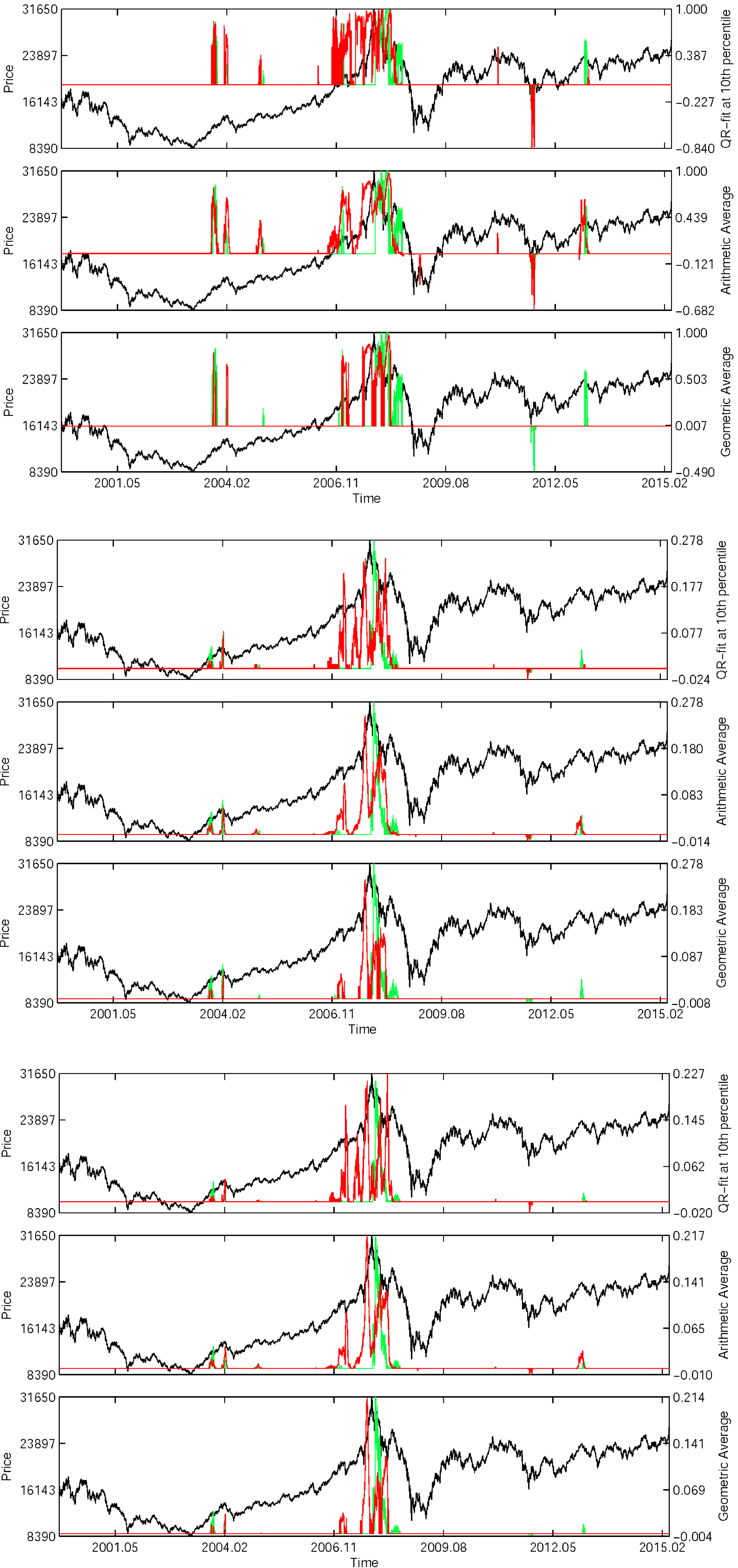

Supplement: S11 Fig — Same as S1 Fig. (TIF) [file pone.0165819.s011.tif]

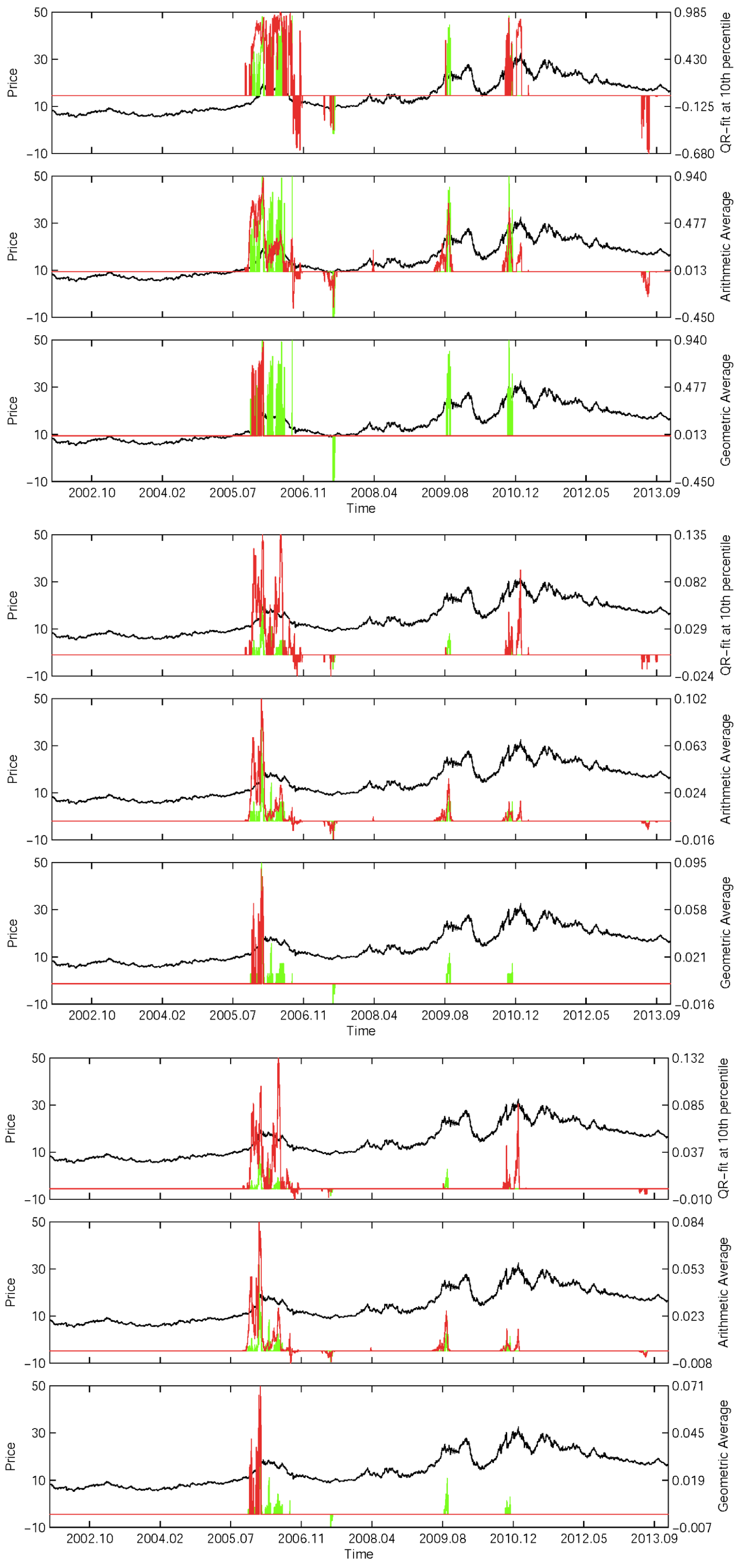

Supplement: S12 Fig — Same as S1 Fig. (TIF) [file pone.0165819.s012.tif]

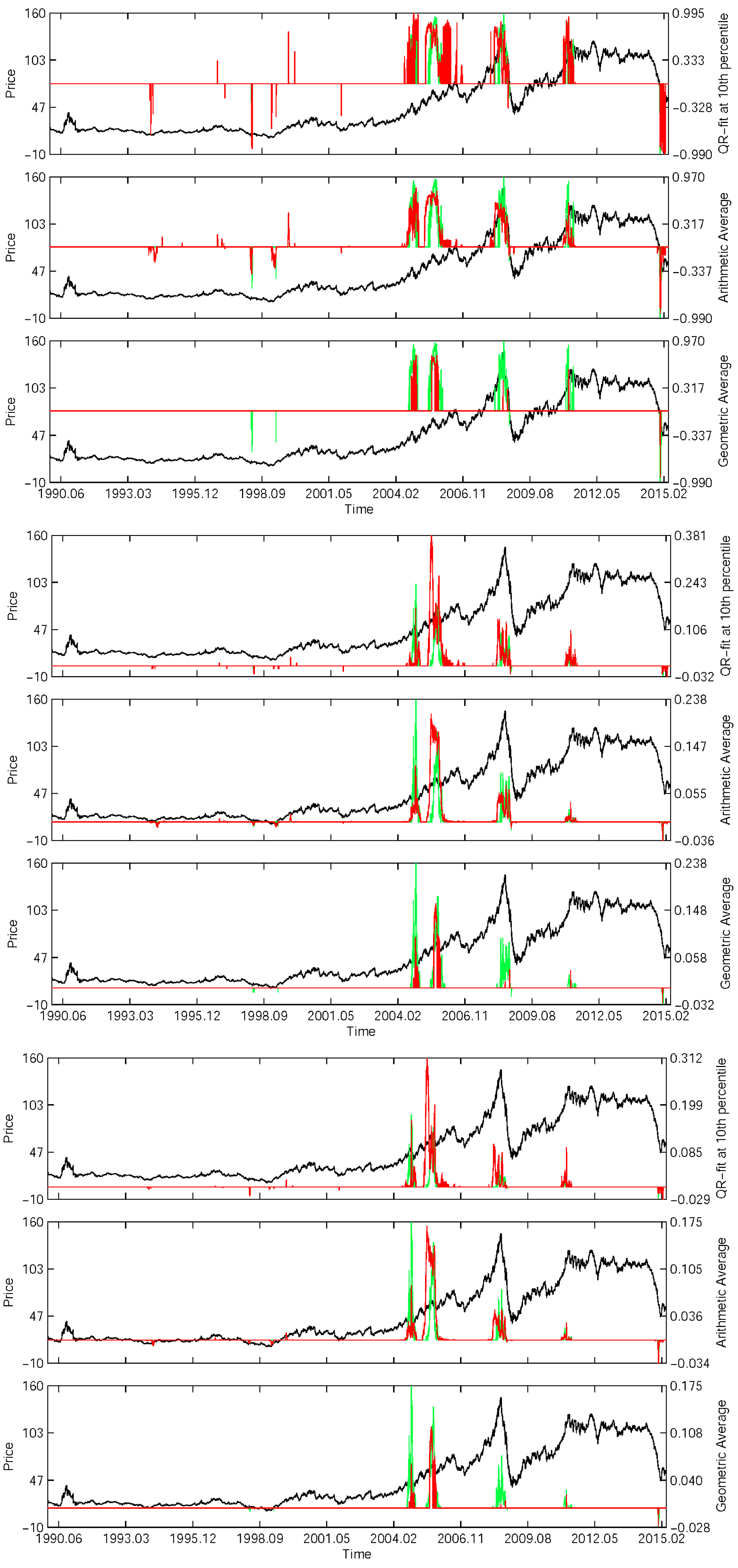

Supplement: S13 Fig — Same as S1 Fig. (TIF) [file pone.0165819.s013.tif]

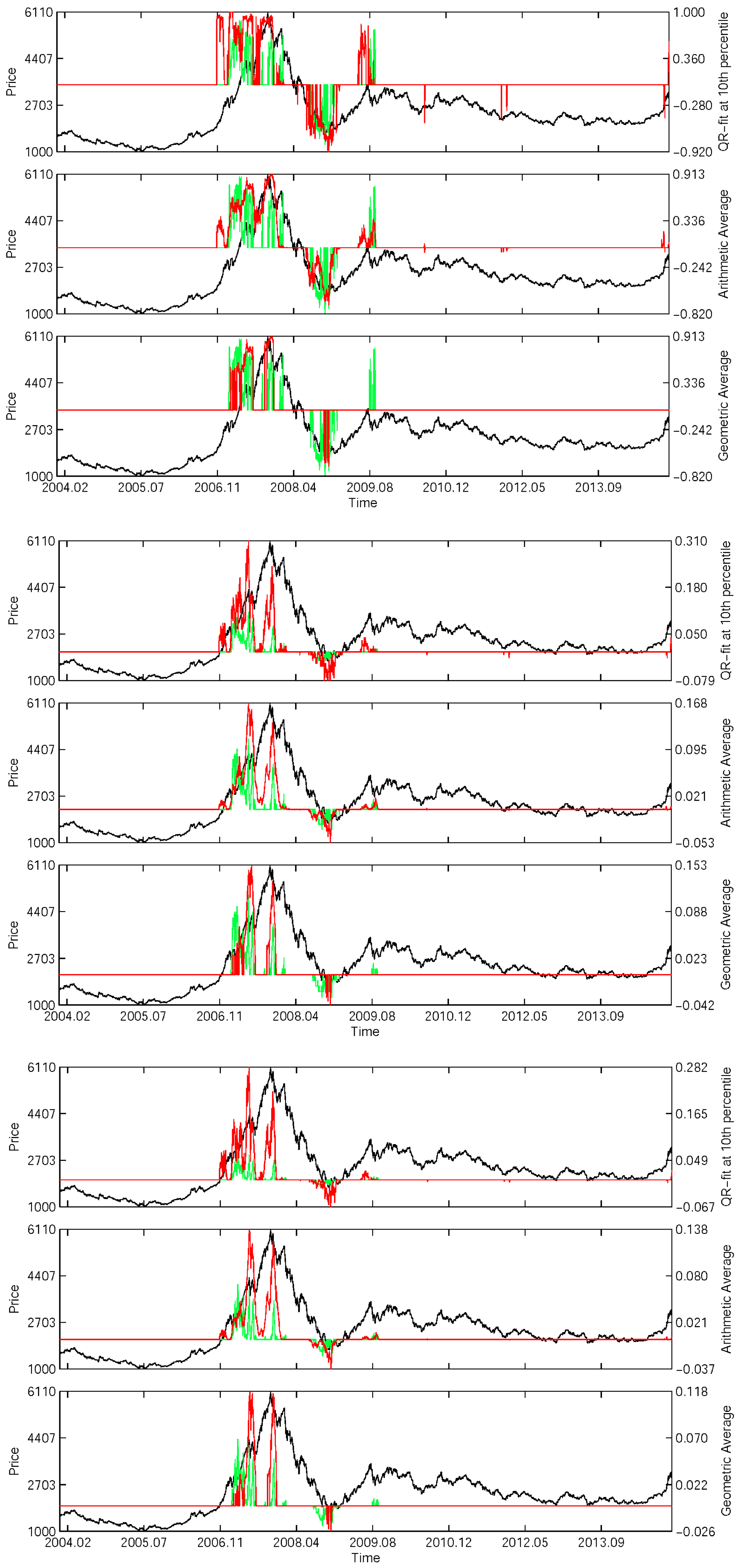

Supplement: S14 Fig — Same as S1 Fig. (TIF) [file pone.0165819.s014.tif]

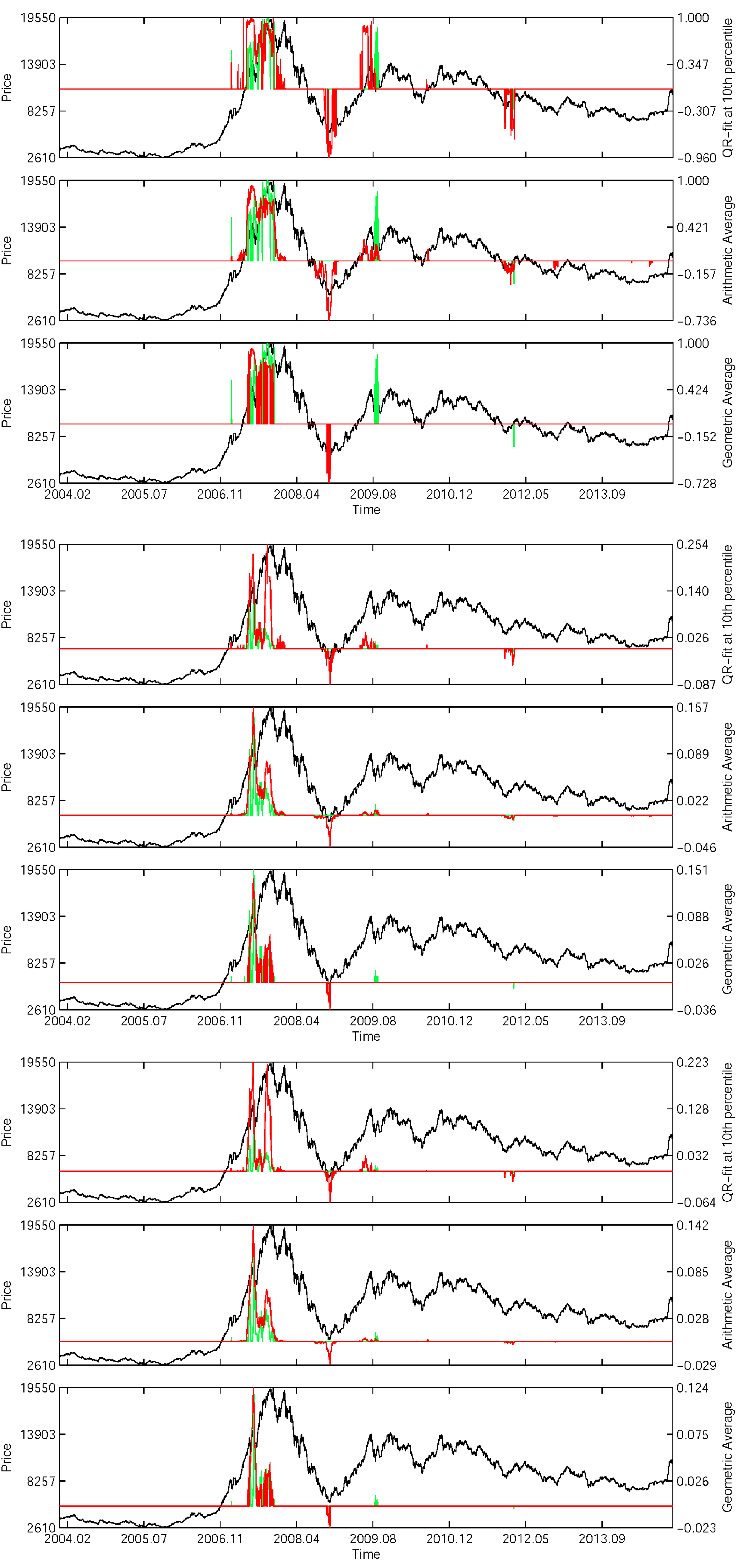

Supplement: S15 Fig — Same as S1 Fig. (TIF) [file pone.0165819.s015.tif]

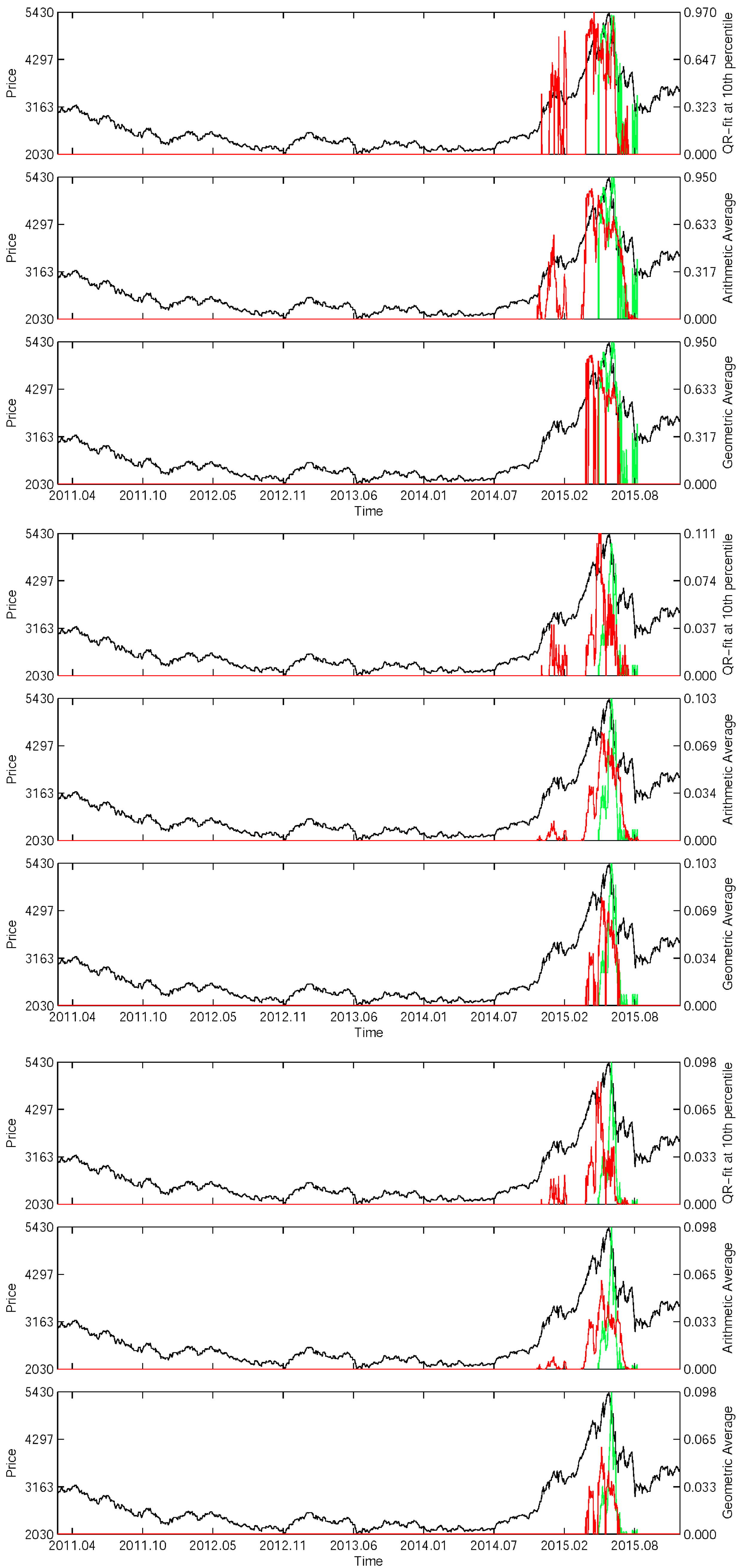

Supplement: S16 Fig — Same as S1 Fig. (TIF) [file pone.0165819.s016.tif]
